# Functionally Graded Materials by Wire Arc Additive Manufacturing: Material-Pair Compatibility and Spatial Mechanical Characterisation—A Systematic Review

**DOI:** 10.3390/ma19163379

**Published:** 2026-08-08

**Authors:** Filipa G. Cunha, Telmo G. Santos, José Xavier

**Affiliations:** 1 Research and Development Unit for Mechanical and Industrial Engineering (UNIDEMI), Department of Mechanical and Industrial Engineering (DEMI), NOVA School of Science and Technology, NOVA University Lisbon, 2829-516 Caparica, Portugaltelmo.santos@fct.unl.pt (T.G.S.); 2 Intelligent Systems Associate Laboratory (LASI), Campus Azurém, 4800-058 Guimarães, Portugal

**Keywords:** functionally graded materials, additive manufacturing, inverse identification methods, mechanical characterisation, wire arc additive manufacturing

## Abstract

Functionally Graded Materials (FGMs) vary in composition and properties for tailored structural performance. Additive Manufacturing (AM), particularly Wire Arc Additive Manufacturing (WAAM), offers a scalable route to metallic FGMs, but manufacture and mechanical qualification remain disconnected. Scopus and Web of Science Core Collection were searched for eligible publications up to and including 31 July 2026. The review includes 176 studies, and every conclusion is graded by a review-specific certainty scheme and bounded to this corpus. The review connects FGM manufacture with full-field inverse identification of spatially varying properties. Evidence indicates interface-defect risk depends on metallurgical compatibility and the composition path. Intermetallic-forming or thermally mismatched pairs remain vulnerable despite process optimisation. Stainless-steel–Ni-superalloy combinations are more frequently reported as sound but only within the composition intervals validated in the cited builds: this apparent advantage reflects unequal study numbers and cracking in specific composition windows. A four-stage sequence—screening phase stability and thermal-expansion mismatch before optimising deposition parameters, then validating the complete composition path—is proposed as an evidence-informed framework rather than a validated decision map. Conventional tests generally provide averaged or location-specific properties rather than a continuous constitutive gradient. Digital image correlation coupled with inverse identification methods can enable the determination of spatially varying properties from a heterogeneous test. However, no experimental study identified a spatial constitutive law across a deliberate WAAM compositional gradient.

## 1. Introduction

Functionally Graded Materials (FGMs) replace an abrupt interface with a prescribed spatial variation of composition, microstructure or porosity [1,2,3,4,5,6,7]. This design can redistribute stress and balance competing functions [8,9,10,11,12], while analogous gradients in bone, teeth, bamboo and insect structures illustrate how spatial organisation can control performance [13,14,15,16,17,18]. The engineering question is therefore not whether heterogeneity is present but whether the manufacturing route can realise the intended gradient without creating a weaker transition region.

Additive Manufacturing (AM) can realise such variations within a near-net-shape component. Among metallic AM routes, Wire Arc Additive Manufacturing (WAAM) is distinguished by high deposition rate, comparatively inexpensive wire feedstock and the possibility of varying the relative feed rates of two wires during deposition [19]. Demonstrations include Fe-Al [20], Fe-Ni [21], Ti-Al [22], Ni-Ti [23], Cu-Al [24], dissimilar steels [25] and stainless-steel–Ni-superalloy combinations [26]. These demonstrations do not, however, establish that every end-member pair or every intervening composition is weldable. Dilution, phase formation, differential thermal contraction and repeated thermal cycling can make the graded region less reliable than either parent alloy.

Qualification poses a related problem. A tensile property assigned to a nominal location, or averaged over a gauge length, does not constitute a spatial constitutive law. Full-field measurements coupled with inverse identification can instead use heterogeneous deformation to estimate material parameters that vary with position [27,28]. The practical transfer of methods developed for homogeneous metals and welded joints to a deliberately graded WAAM alloy nevertheless remains unproven.

This systematic review connects two questions that have usually been treated separately: (i) how can compositionally graded metallic components be designed and manufactured by WAAM; and (ii) how can their spatially dependent mechanical properties be determined from a limited number of heterogeneous tests? Using Preferred Reporting Items for Systematic Reviews and Meta-Analyses (PRISMA) 2020 [29], the review first maps material-pair and composition-path evidence, then distinguishes mechanical measurements already demonstrated on graded builds from inverse methods demonstrated only on related heterogeneous specimens. This structure permits the certainty and transfer limits of each conclusion to be stated explicitly. Section 2 reports the review methods, Section 3 presents the evidence, Section 4 develops the compatibility and characterisation frameworks, and Section 5 separates established findings, recommendations and hypotheses.

## 2. Materials and Methods

### 2.1. Search Strategy

The systematic review was conducted with reference to the JBI Manual for Evidence Synthesis [30] and is reported in accordance with PRISMA 2020 [29]. The protocol was registered in the Open Science Framework (OSF; https://doi.org/10.17605/OSF.IO/E3TGV). Searches of Scopus and the Web of Science Core Collection covered the period from database inception to the eligibility cut-off date of 31 July 2026. Scopus searched TITLE-ABS-KEY; Web of Science used the closest practical translation, Topic search TS. Table 1 reports the shared query bodies; the database-specific field wrappers, operational calendar-year bounds and query result counts are reported in Supplementary Table S2. No separate terminology-sensitivity query was counted among the nine searches.

The searches yielded 1649 raw occurrences. Exact DOI, database identifier and normalised-title consolidation removed 767 repeated query/database occurrences and produced 882 canonical records; 629 occurred in both databases. The raw exports, checksums, occurrence-to-record mapping, duplicate decisions and screening logs were retained in the review archive. The exports contain Article/Review records from Scopus and journal records from Web of Science. Exported language fields were blank, so English eligibility was verified from title/abstract and, where retrieved, the full text rather than claimed as a database-interface filter.

A reference-list, citation and publisher-record sensitivity check identified Han et al. [31], Liu et al. [32] and Wang et al. [33]. None was present in the 882-record database pool. Because all three contribute evidence and satisfy the same eligibility criteria, they are now reported in the PRISMA “other methods” branch rather than being discussed outside the denominator: three identified, three retrieved and assessed, no full-text exclusions and three included studies.

### 2.2. Inclusion and Exclusion Selection Criteria

Eligibility was defined for three linked evidence streams: (i) experimental manufacture or mechanical characterisation of graded materials used to establish general process and testing principles; (ii) experimental metallic AM, WAAM or dissimilar-material transitions reporting process–structure–property evidence; and (iii) inverse identification of spatially varying or local properties in FGMs, welded metals, AM materials or closely related heterogeneous specimens. Peer-reviewed journal articles in English were eligible when they reported experimental data or validated an identification method with experimental or synthetic full-field data. Numerical proof-of-concept studies were retained only in the method-validation stream and were distinguished from experimental demonstrations. Conference papers, book chapters, purely numerical gradient-design studies without method validation, articles without relevant mechanical or microstructural evidence, and non-metallic applications unrelated to grading or method transfer were excluded. Foundational books and standards could be cited for context without being counted among the 176 included studies. Review papers were not allowed to contribute primary evidence to a synthesised claim; where retained in the scope map, they were classified as contextual and were not appraised against primary-study domains. Avril et al. [34] was excluded during the full-text screen and is cited only as an external contextual source. Validated primary method studies using heterogeneous fields on homogeneous metallic specimens were eligible as indirect method-transfer evidence but were not allowed to support a claim about a manufactured gradient.

### 2.3. Critical Appraisal and Evidence-Certainty Assessment

Relevance was determined during eligibility screening and was not conflated with methodological completeness. Because the evidence comprised both manufacturing experiments and inverse-identification studies, the narrative appraisal used two design-specific five-domain frameworks. Manufacturing evidence was examined for: (M1) material and process specification; (M2) verification of the intended composition path; (M3) microstructure or phase identification; (M4) mechanical-test reporting, including sampling and repeatability; and (M5) support for the process–structure–property interpretation. Inverse-identification evidence was examined for: (I1) specimen, loading and boundary-condition reporting; (I2) full-field measurement and uncertainty reporting; (I3) constitutive-model and parameter definition; (I4) objective function, sensitivity, identifiability or regularisation; and (I5) independent validation. Appraisal was not used as a post hoc exclusion criterion, and the domains were not combined into a summary score.

#### 2.3.1. Application and Reporting of the Appraisal Domains

Each study contributing evidence to a synthesised claim was rated against the relevant five domains on a four-level qualitative scale: met, the domain is explicitly and completely reported; partly met, the domain is reported but is incomplete, restricted to part of the specimen or path, or reported without the accompanying quantitative detail; not met, the domain is applicable to the study design but is not reported; and unclear, the domain could not be resolved from the fields extracted for this review. The last category is a statement about the present extraction and not a judgement that the primary study failed to report the item. The completed study-level ratings are given in Supplementary Tables S3 and S4. The domains are not weighted or summed, because the corpus contains no common outcome measure against which weights could be calibrated; they are used instead as named reasons for downgrading a claim as set out below.

Supplementary Table S1 provides the study-level accounting against which the corpus total can be checked. It lists all 256 works cited in the review, identifies exactly 176 as included studies and distinguishes the other 80 contextual or non-study references. Of the 176 included studies, 94 contribute manufacturing or inverse/full-field evidence to a synthesised claim and are appraised in Supplementary Tables S3 and S4; the remaining 82 serve scope-mapping or contextual roles for which M1–M5 and I1–I5 are not applicable.

#### 2.3.2. Evidence Tiers

Because manufacturing evidence and method-transfer evidence differ in directness, every included study contributing to a claim was also assigned to one of four tiers: (E1) direct evidence from deliberate compositional gradients produced by wire-fed arc processes; (E2) supporting evidence from other metal additive-manufacturing processes, from bimetallic or sequentially deposited arc builds without a graded composition path, and from arc processes requiring a second, non-wire feedstock; (E3) indirect experimental evidence from welds, architectured materials, local-gradient specimens or heterogeneous tests of otherwise homogeneous materials; and (E4) numerical or synthetic-data proof of concept without an accompanying physical identification experiment. The tier is reported for every evidence-bearing study in Supplementary Tables S3 and S4 and is carried through the synthesis tables and Section 4 and Section 5. Tier E1 alone can support a claim about WAAM gradients; tiers E2–E4 can support a claim about a method or a mechanism, but transfer to a deliberate WAAM gradient is treated throughout as a hypothesis rather than as evidence.

#### 2.3.3. Certainty of Cross-Study Claims

Certainty was then assigned to each cross-study conclusion. The scheme below is a review-specific device for organising evidence, developed for this corpus. It is not a validated certainty-of-evidence instrument, it was not derived from GRADE or from any formal risk-of-bias tool, and its categories should not be read as equivalent to those systems. It is reported so that the basis of each judgement is inspectable, not so that the resulting grades can be pooled with grades produced elsewhere.

A provisional level was first set from directness and replication: high required reproduction in at least three independent material systems at tier E1 with consistent phase, defect and mechanical evidence; moderate required two such systems, or several studies of one system, with no major unexplained contradiction; and low/preliminary applied to a claim resting on one system, one or two reports, tier E4 evidence, or transfer from another material class. The provisional level was then revised by three explicit rules, each of which was applied before the final grade was recorded.

- Appraisal downgrade. If the domain most critical to the claim was rated partly met, not met or unclear in the majority of the contributing studies, the level was reduced by one. Which domain is critical depends on the claim: M2 for any statement about a composition path, M3 for a statement about phase formation, M4 for a statement about mechanical response, and I2, I4 and I5 for statements about identified parameters. Study count therefore cannot raise a claim past the completeness of the evidence supporting it, and three incompletely reported systems do not outrank two thoroughly reported ones.
- Directness downgrade. A claim about deliberate WAAM gradients supported mainly by tier E2–E4 evidence was reduced by one level, whatever the number of contributing studies.
- Inconsistency downgrade. Contradictory findings that the primary reports do not explain by a stated difference in composition interval, process window or thermal history reduced the level by one. Contradictions that the reports do explain in those terms were retained as a boundary condition on the claim rather than as a downgrade, and the boundary is stated in the claim itself.

Two worked examples show how the rules operate. The claim that steel–Inconel paths can produce sound transitions begins at high because more than three systems—most of them tier E1, the remainder sequentially deposited tier-E2 builds—report sound transitions. Cracking is nevertheless reported in particular intermediate windows in the same alloy family, and the primary reports attribute this to identified composition intervals rather than to unexplained scatter; the contradiction therefore restricts the claim to the validated intervals instead of downgrading it. M4 is, however, only partly met across the group—replicate counts and scatter are rarely reported, and one of the systems reports no tensile data at all—so the appraisal rule applies and the recorded level is moderate. Conversely, the claim that thermal-expansion mismatch contributes to refractory-pair cracking begins at low/preliminary on replication alone since it rests on a single within-study Ta-Mo versus Mo-W contrast; M3 is met but the design does not isolate thermal expansion from phase and processing effects, so no upgrade is available and the level is retained. The claim-level evidence assessment records the evidence base, tier, consistency, the governing appraisal limitation and the final level for each principal conclusion.

### 2.4. Study Selection

Filipa G. Cunha screened the 449 records that entered title-and-abstract screening under the registered protocol, and José Xavier then re-applied the criteria to every record with the first decision visible. The dual-database search was handled as an incremental screen. The confirmation table retained the advisory fields used to prepare the records. Record matching and deduplication were performed with purpose-written scripts, and the draft dispositions were prepared with a generative artificial intelligence assistant; both are automation aids only. The advisory entries were held in separate columns from the reviewers’ own entries and were not substituted for the reviewers’ decisions, which departed from the advisory disposition on two records subsequently resolved under the confirmed English-language eligibility rule. Separate FC and JX full-text decision files were completed. A single difference between them was resolved by consensus to Include after returning to the full text. Four reports were excluded with one primary reason each, while five unavailable reports remained “not retrieved” and were not reclassified as eligibility exclusions.

Data extraction retained the material pair, process, realised gradient or heterogeneous zone, characterisation method, constitutive model, validation and principal outcome. Extraction was performed in a single pass, without a duplicated per-item log. The extracted rows were rechecked against the primary reports when Supplementary Table S1 was compiled, a citation-context classification of every cited work prepared with the same automation aids, and checked against where each work is cited in the manuscript. Two entries changed at that stage: one study moved from evidence-bearing to contextual and left Supplementary Table S4, and one I2 rating moved from met to partly met. Both changes are reflected in the tables reported here. The M1–M5 or I1–I5 domains and E1–E4 tiers were applied to every study contributing evidence to a synthesised claim, and the resulting study-level ratings and governing notes appear in Supplementary Tables S3 and S4. Directness was preserved throughout: welded, oxidation-induced, explosively bonded or homogeneous method-validation specimens were not reclassified as deliberate WAAM composition gradients.

## 3. Results

### 3.1. Publication Overview

**Figure 1:**
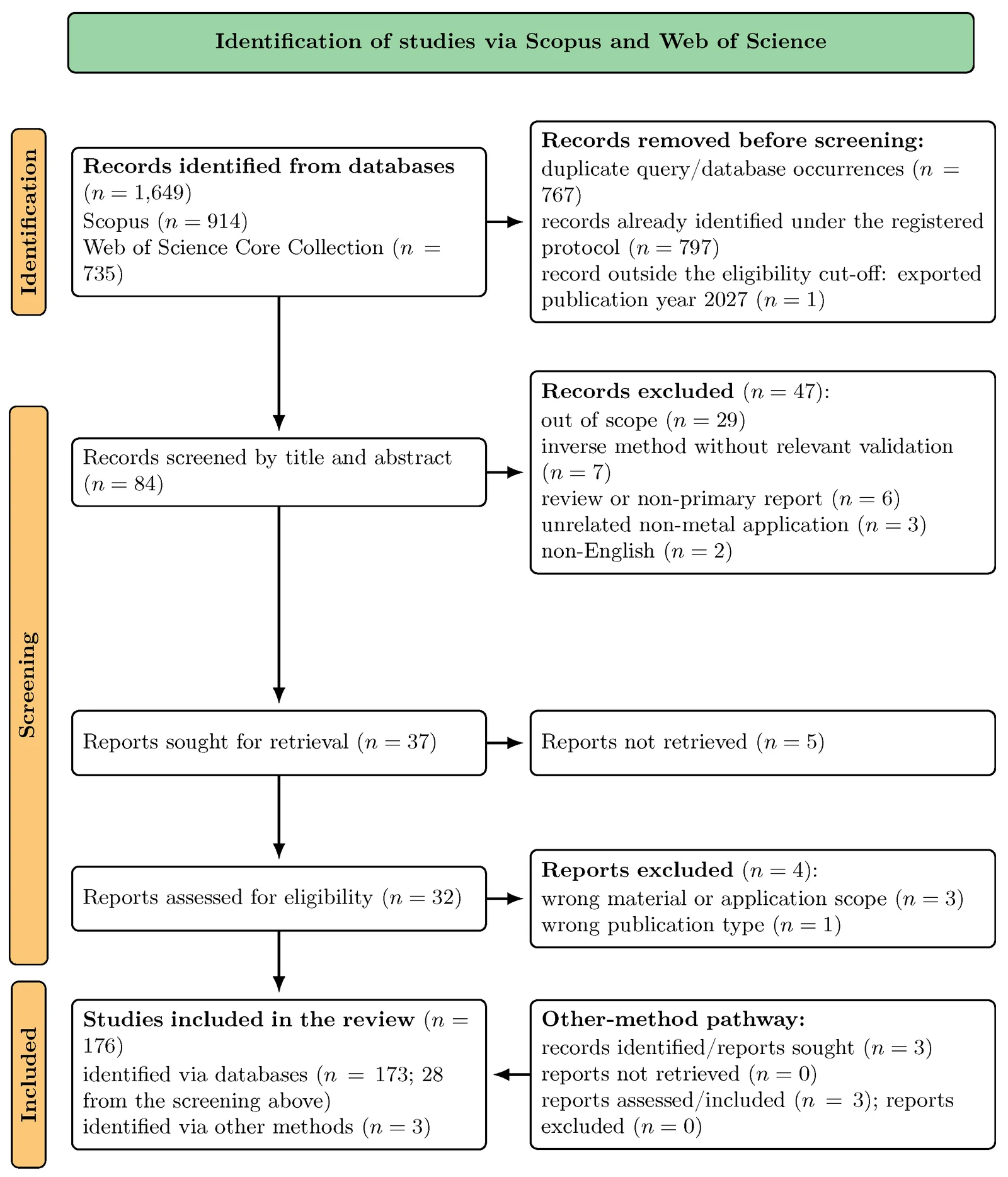
PRISMA 2020 flow diagram for the consolidated review, adapted from the official template for database, register and other-method searches [29]. Eligible publications were considered up to and including 31 July 2026.

The PRISMA flowchart in Figure 1 presents the consolidated dual-database review through 31 July 2026. Record, report and study counts are labelled separately.

**Figure 2:**
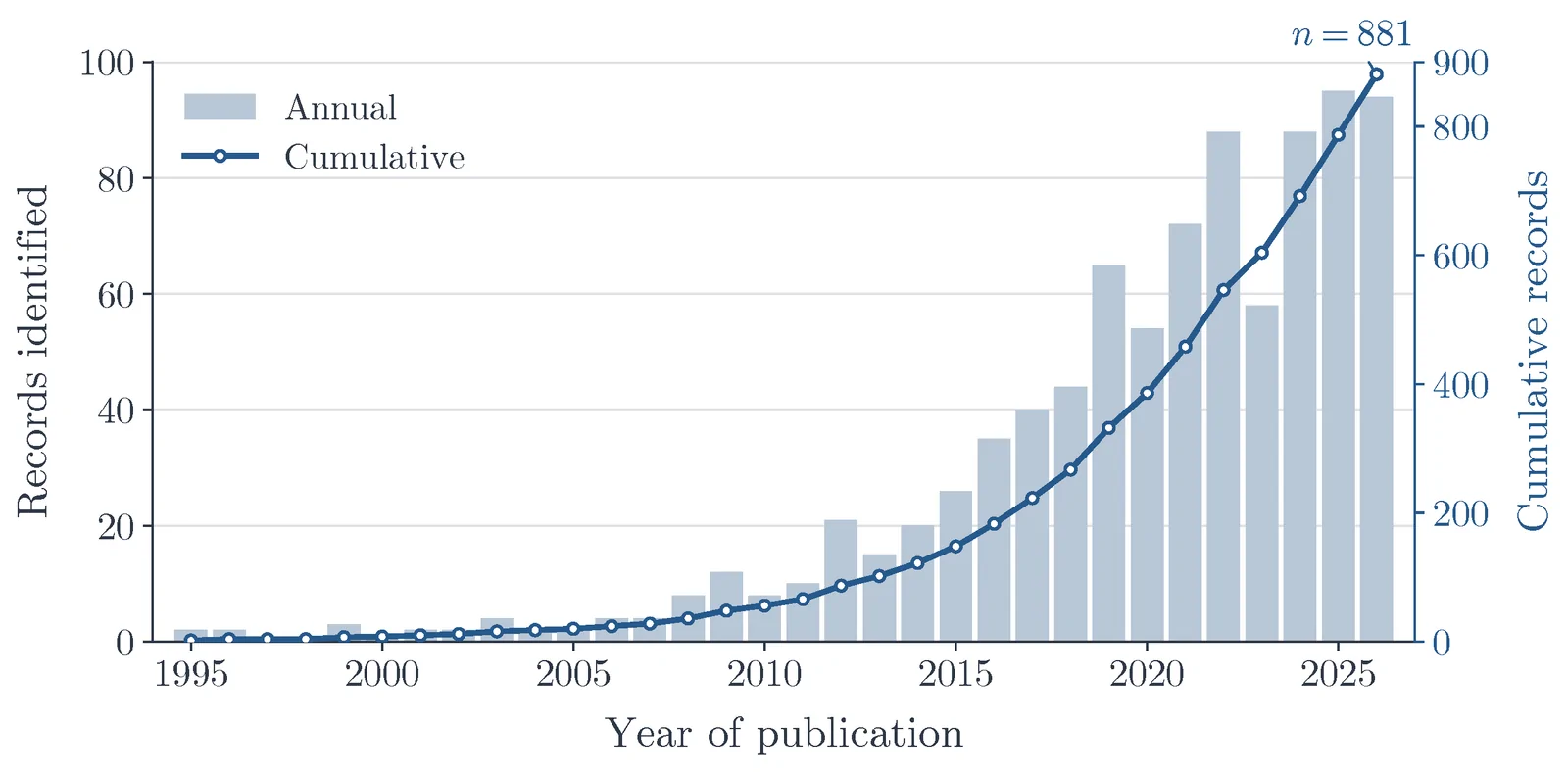
Publication-year profile of the 881 unique records identified by the dual-database search of Scopus and Web of Science Core Collection, after removal of the 767 duplicate query and database occurrences from 1649 raw occurrences. Bars give records per year and the line their cumulative total. The 2026 search period ended on 31 July 2026, so its bar represents a partial year.

The searches identified 1649 database occurrences: 914 from Scopus and 735 from Web of Science Core Collection. Removing 767 duplicate query/database occurrences produced 882 unique records. Of these, 881 fell within the eligibility window. The date-eligible records are weighted towards recent work: 70% were published from 2019 onwards, the median publication year is 2021, and the annual count peaks at 95 in 2025 (Figure 2). The 2026 bar covers only the seven months to the eligibility cut-off and therefore understates that year. Of the 881 records, 797 had already been screened under the registered protocol and the remaining 84 were screened by title and abstract: 47 were excluded and 37 reports were sought, of which 5 could not be retrieved. Thirty-two full texts were assessed and four were excluded, three for the wrong material or application scope and one for the wrong publication type. Screening and full-text assessment across the pool yielded 173 included database studies. The reference-list, citation and publisher-record branch contributed three further eligible studies, all retrieved and assessed and none present in the 881-record database pool so that the corpus contains 176 studies (Figure 1).

The following subsections are organised to systematically address the two central questions of this review. Section 3.2 addresses the first question by examining FGM types, conventional and AM-based manufacturing methods, applications, and WAAM-specific developments. Section 3.3 directly addresses the second question by reviewing inverse identification methodologies for determining spatially varying mechanical properties.

### 3.2. Functionally Graded Materials

FGMs are classified by what varies spatially and by whether the variation is continuous or stepwise. This distinction matters for WAAM: changing the relative wire-feed rates can create a nominally continuous composition path, whereas changing wire between layers creates a stepwise path whose local composition is subsequently smoothed by remelting and dilution.

#### 3.2.1. Types of Functionally Graded Materials

The three established classes are composition, porosity and microstructure gradients [35,36]. Table 2 retains this classification as a scope map. The compatibility synthesis below concerns compositionally graded metal–metal systems; porosity- and microstructure-graded examples are used only to compare manufacturing or characterisation methods.

#### 3.2.2. Conventional Methods of Manufacturing Functionally Graded Materials

Table 3 summarises the established gas-, liquid- and solid-phase routes [3,37,38,39]. They provide useful control of coatings, porosity or simple layered geometries, but generally offer less freedom to vary a metal–metal composition path within a large three-dimensional component. The table is retained as a concise baseline for the AM comparison rather than as a separate review of conventional processing.

The review also retained experimental graded-material studies outside the metal–metal WAAM subset. These include centrifugally graded particle composites, sintered W–steel and Al–Zn systems, Cu–Ni–Si/graphite and hydroxyapatite–Ti gradients, polyester indentation, hybrid composite rods and AM microstructured connectors [59,60,61,62,63,64,65,66]. They establish the diversity of gradient manufacture and local testing, but they are not used to infer metallurgical compatibility for fusion-deposited metal–metal paths.

#### 3.2.3. Additive Manufacturing of Functionally Graded Materials

Of the seven ISO 17296-2:2015 AM categories [67], material extrusion, powder-bed fusion, directed-energy deposition and sheet lamination have been used to produce FGMs [2,68,69,70]. Demonstrations outside WAAM include locally controlled material extrusion, graded powder extrusion, microstructure grading by laser powder-bed fusion and selective laser sintering of particulate-filled polymers [71,72,73,74]. The review does not estimate process shares from the search exports: the queries overlap intentionally, and repeated query/database occurrences are not independent publications. The comparison therefore focuses on feedstock, spatial resolution, build scale and evidential directness.

Material extrusion serves chiefly polymer, polymer–ceramic and paste-based gradients—alumina–zirconia, graded ABS, direct-ink-written B_4_C–SiC and PLA–hydroxyapatite scaffolds [75,76,77,78]—and establishes compositional control at modest cost without transferring to fusion processing of structural metal–metal gradients.

The three metallic routes divide by resolution, scale and feedstock. Powder Bed Fusion (PBF) offers the finest geometrical and microstructural resolution—graded-porosity implants and lattices, and selectively deposited IN625–CuCrZr joints [79,80,81,82,83]—but its build envelope and powder handling place it outside the large-component design space that motivates WAAM. Directed Energy Deposition (DED) is directly relevant because wire or powder feed rates can be varied during deposition; laser DED has produced SS316L–Inconel 625, AISI 316L–Co-superalloy and H13–Cu gradients [84,85,86]. Ultrasonic sheet lamination has joined aluminium, copper, stainless-steel and Ni-Ti foils into layered gradients without bulk melting but is restricted to sheet feedstock [87,88,89]. WAAM occupies the high-deposition-rate, wire-fed part of the DED family and is examined separately below. Evidence from all three routes is tier E2 throughout this review: it can support a claim about a mechanism but not about a WAAM gradient.

The comparison therefore reduces to feedstock compatibility and the trade-off between resolution and scale. Extrusion serves mainly polymers and pastes; PBF provides resolution within a smaller build envelope; sheet lamination produces layered assemblies; and DED accepts dissimilar metallic feeds at component scale. WAAM is the DED variant most clearly aligned with large metal–metal FGMs. This feedstock-and-scale alignment, rather than a database-frequency estimate, motivates the following section.

#### 3.2.4. Areas of Applications of Functionally Graded Materials

**Figure 3:**
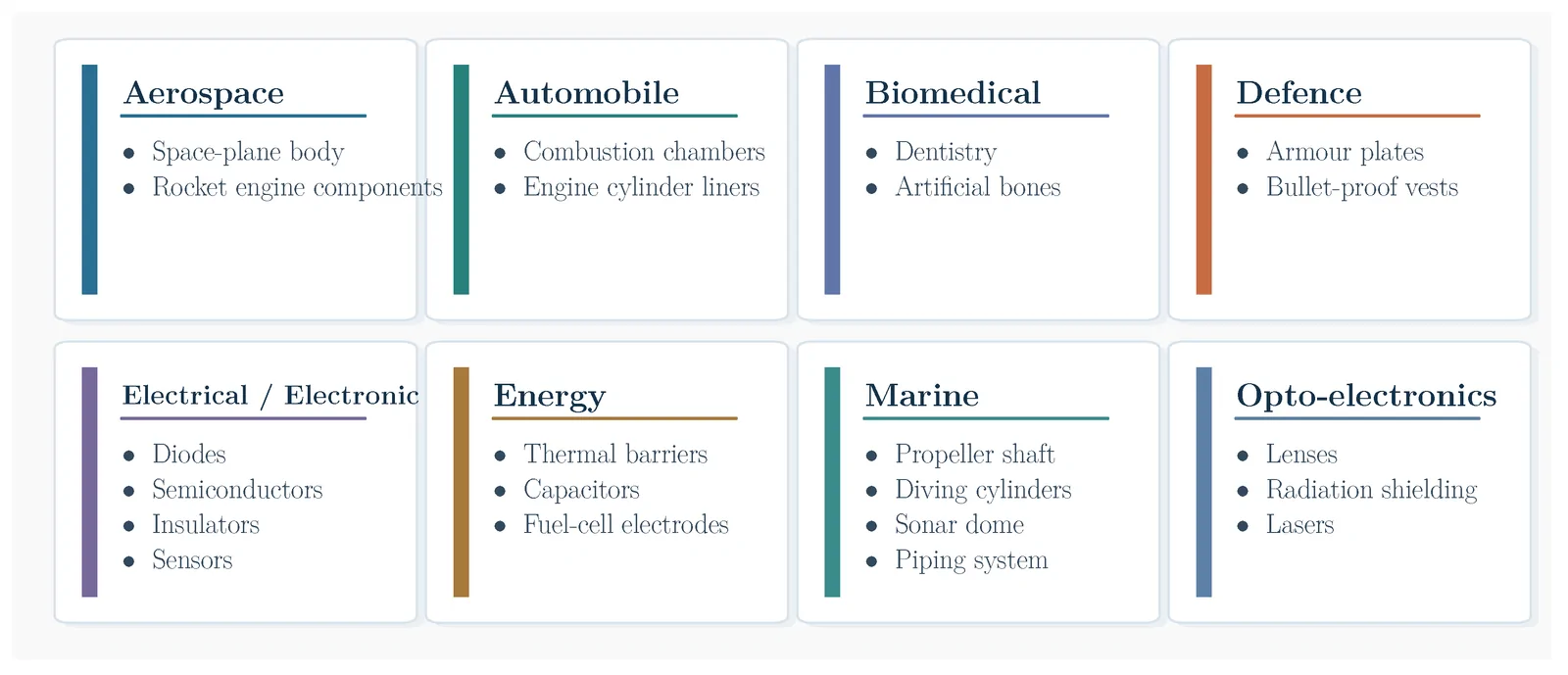
Applications of FGMs across engineering sectors.

The broad application landscape is retained in Figure 3 only to delimit the WAAM-relevant subset. WAAM is restricted to fusible metallic wire feedstocks and therefore cannot produce the metal–ceramic or semiconductor gradients that dominate thermal-barrier, biomedical and optoelectronic applications. Its defensible application space comprises large aerospace, energy, marine and defence structures; bimetallic pipework; and local corrosion- or wear-resistant transitions. The WAAM-specific synthesis is confined to this metal–metal subset.

#### 3.2.5. Functionally Graded Materials Using Wire Arc Additive Manufacturing

WAAM couples an arc heat source with wire feedstock and is attractive for large graded metallic structures because two independently controlled wires can vary melt-pool composition during deposition [19]. Its high deposition rate is accompanied by limited geometric resolution, repeated thermal cycling, dilution, porosity, residual stress and possible brittle phase formation. Table 4 maps the reported composition paths; the text below retains only outcomes needed to compare path compatibility and mechanical characterisation.

##### Fe-Al

Twin-wire GTAW-based WAAM varied Al from 15 to 55 at.% by changing the Fe/Al feed ratio [20]. A related Fe_3_Al wall showed nearly uniform composition and microhardness but different longitudinal and transverse tensile responses, attributed to epitaxial grain growth [90]. Composition control therefore did not remove process-induced mechanical anisotropy.

##### Fe-Ni

Twin-wire WAAM produced Fe_3_Ni–FeNi gradients containing 20–50% Ni [21,91,92]. Microscopy and neutron diffraction resolved the phase and anisotropic lattice evolution during deposition and subsequent heating. These studies establish process feasibility and phase evolution, but do not provide a spatial mechanical constitutive law.

##### Ti-Al

Dual-wire GTAW-based WAAM graded pure Ti to Ti–50% Al by controlling the two feed rates [22]. Related γ-TiAl studies show that interpass temperature changes the thermal history and resulting microstructure [93,94]. The evidence demonstrates compositional control, but the sensitivity of Ti-Al phase formation to the thermal cycle limits transfer between process windows.

##### Ni-Ti

WAAM NiTi gradients were predominantly austenitic at room temperature and exhibited tensile superelasticity [23]. A related wall study linked the layerwise thermal history to variations in microstructure, phase transformation and mechanical response [95]. These results establish functional behaviour, while also showing that a nominally fixed composition can retain a process-induced property gradient.

Bulla et al. [96] add a twin-wire DED-arc study of equiatomic NiTi in which the target composition was fixed rather than deliberately graded. Local thermal cycling changed the lattice and microstructure along the build and promoted silicon-rich brittle precipitates. The study is consequently tier E2 support for thermal-history sensitivity in twin-wire arc deposition, not an additional demonstration of a programmed Ni-Ti composition gradient.

##### Cu-Al

Dong et al. [24] produced Cu-Al alloys by twin-wire WAAM with a GTAW heat source, a route of interest to shipbuilding because these alloys combine wear and corrosion resistance; conventionally they are made by powder metallurgy. Argon shielding protected the copper and aluminium feedstock during deposition. The gradient was set by feeding a 99.9% copper wire and a 1080-grade aluminium wire, both 0.9 mm in diameter, and the intermetallic phases CuAl_2_ and Cu_9_Al_4_ were identified in the deposited material.

Extending the Cu-Al system, Cunha et al. [97] assembled a library of Cu-CuAl compositions produced by WAAM using both gas metal arc welding (GMAW) and Gas Tungsten Arc Welding (GTAW) variants, and characterised them by hardness testing, eddy-current measurements and tensile testing supported by Digital Image Correlation (DIC). Hardness and ultimate tensile strength increased with the CuAl fraction, whereas a higher Cu content improved ductility, so that the compositional library maps a continuous strength–ductility trade-off across the gradient. This approach is notable in the present context because it couples a WAAM compositional gradient directly with full-field (DIC) measurement, anticipating the inverse-characterisation methods discussed in Section 3.3.

##### Ta-Mo-W

Marinelli et al. [98] deposited two graded refractory transitions—tantalum to molybdenum, and molybdenum to tungsten—on a tantalum substrate, and examined microstructure, hardness and chemical composition. A dense crack network formed at the Ta-Mo interface, attributed to the mismatch in thermal expansion coefficient between the two metals. Composition and hardness varied markedly through the first Mo-W layers, reflecting the difference in melting point and the rapid solidification of the melt pool, but no cracking was observed between the molybdenum and tungsten layers, whose thermal expansion coefficients are similar. The authors identify turbine blades, rocket nozzles and nuclear reactor components as potential applications.

##### HSLA-Cu-Al

Rodrigues et al. [26] produced a copper-alloy–High Strength Low Alloy (HSLA) steel FGM by Twin-Wire Arc Additive Manufacturing (T-WAAM). The pairing is industrially attractive because it combines high thermal and electrical conductivity with wear resistance and good mechanical properties, but copper and steel differ in thermal expansion coefficient, melting temperature and crystal structure. Optical and scanning electron microscopy and high-energy synchrotron X-Ray Diffractometer (XRD) revealed a smooth gradient in hardness and electrical conductivity along the build height, with a tensile strength of 690 MPa and an elongation of 16.6%.

##### Inconel 625–HSLA

Four studies examined gradients between Inconel 625 and HSLA steel [99,100,101,102]. Electromagnetic stirring reduced primary dendrite spacing by 22.6% and increased tensile strength from 512 MPa to 563 MPa; an optimised transition strategy avoided a weak composition interval; and in situ heat treatment increased build-direction strength to 602(10) MPa. These are distinct mitigation mechanisms and should not be treated as interchangeable process optimisation.

##### Ti6Al4V–Inconel 625

Han et al. [103] graded Ti6Al4V to Inconel 625 across the full composition range by dual-wire WAAM, reporting an average ultimate compression strength of 1390.25 MPa at 10.96% strain for vertical samples. The mechanical evidence is confined to one orientation and one loading mode, so the build demonstrates that the path can be deposited rather than that it is mechanically sound throughout.

##### Ti6Al4V–Stainless-Steel 316L

A dual-wire gradient spanning 5–65 at.% Fe developed cracking at 50 at.% Fe that extended into the 35 at.% Fe layer [104]. XRD identified Fe_2_Ti in the affected compositional region, and the maximum hardness, approximately 945 HV0.2, occurred at 60 at.% Fe. This result shows that successful deposition of the endpoint alloys and high local hardness do not guarantee a crack-free continuous composition path under these processing conditions.

##### Inconel 625–Stainless-Steel 316L

Ahsan et al. [105] produced a Bimetallic Additively Manufactured Structures (BAMS) combining Inconel 625 and austenitic stainless steel SS316L by WAAM. Electron microscopy and elemental mapping were used to characterise interfacial crystallographic growth and elemental diffusion, which showed grains elongated in one direction at the interface. Microhardness, tensile testing and fractography gave hardness values of 220–240 HV in both materials and a tensile strength of 600 MPa at an elongation of 40%. Inconel 625 and austenitic stainless steel are widely paired in bimetallic structures for the aerospace, energy, petrochemical, chemical and marine industries.

A later wire-arc DED study by Ahsan et al. [106] deliberately used microstructural and compositional heterogeneity in an SS316L–Inconel 625 face-centred-cubic build to obtain a strength–ductility synergy. Its compositionally heterogeneous architecture and DIC-supported mechanical response strengthen the tier-E2 evidence that local heterogeneity can be engineered in this alloy family. Because the report concerns a heterogeneous/bimetallic architecture rather than identification of a continuous spatial constitutive law, it does not close the inverse-characterisation gap.

Yu et al. [109] compared SS316L–Inconel 625 gradients deposited by dual-wire plasma-arc Additive Manufacturing using layerwise composition changes of 25 wt.% (sample S1) and 100 wt.% (sample S2). Both builds were predominantly austenitic and no cracks or other defects were observed. Sample S1 had its lowest microhardness and tensile strength in the 75 wt.% SS316L region, whereas S2 produced the better tensile response; the two designs also fractured at different positions. The comparison therefore demonstrates that the composition increment changes the local property distribution and tensile response even when both paths remain crack-free.

##### Stainless Steel 308L–Inconel 625

Stainless steel 308L and Inconel 625 have been graded by WAAM in a group of related studies [110,111,112,113]. The pairing is appropriate because the nickel-based alloy provides strength and high-temperature performance, whereas the stainless steel provides corrosion resistance at lower cost. Li et al. [110] graded the composition from 100% SS308L in the base layers to 100% Inconel 625 in the final layers, producing a transition aligned with the direction of torch travel, and characterised composition, microstructure, phases and mechanical properties. A companion study [113] examined the type, location, cause, initiation and propagation of cracks formed during deposition, together with measures to eliminate them. A weakened interval between 10% and 50% Inconel 625 was subsequently strengthened by local Nb alloying, which raised the tensile strength from 385 MPa to 510 MPa and the elongation from 8.3% to 16.9% [111]. Two wire-feeding strategies affecting the geometry, microstructure and mechanical properties of the graded wall were then compared [112].

##### Stainless Steel 304L–Inconel 625

Yoo et al. graded 75% SS304L–25% Inconel 625 to the inverse proportion and compared WAAM with a hybrid LMD–WAAM route [114]. Hardness increased with Inconel content and the hybrid route raised tensile strength by 8%, but it also moved fracture from the SS304L vicinity to an internal composition interface. A higher global strength therefore did not remove the local transition risk.

##### Duplex Stainless Steel–Carbon-Manganese Steel

Marine risers convey crude oil and gas from the wellhead to offshore facilities, and are exposed to a corrosive environment on the outside and to oxidation by the transported hydrocarbons on the inside. Chandrasekaran et al. [25] produced a riser FGM by WAAM, with duplex stainless steel on the inside of the conduit and carbon-manganese steel on the outer surface. As is common in WAAM-produced FGMs, material diffused between layers to form a coalescence zone in which the two steels merge.

The build was characterised by X-ray CT, tensile testing, microstructural analysis, EDX, microhardness, fractography and corrosion testing—the most complete characterisation set in the reviewed WAAM evidence—but the coalescence zone is a diffusion product of two sequential deposits rather than a programmed composition path.

##### Low-Carbon Steel–Stainless-Steel 316L

Ahsan et al. [116] built a low-carbon-steel/SS316L bimetallic structure by WAAM and heat-treated it from 800 °C to 1100 °C, characterising it by SEM, EDX and Vickers hardness. Heat treatment moved the failure site from the low-carbon steel to the stainless steel, which shows that post-deposition thermal processing relocates the weakest region of a transition instead of simply strengthening it.

##### Low Alloy Steel–Austenitic Stainless Steel

An interleaved low-alloy/austenitic-stainless-steel wall created repeated interfaces rather than a monotonic composition ramp [117]. XRD indicated a predominantly ferritic graded region, and hardness was greatest in the low-alloy-steel layers. Tensile and fatigue tests demonstrated structural continuity, but the architecture should be distinguished from a continuously graded path.

##### Super Austenitic Stainless Steel SS904L–Hastelloy C-276

Rajesh Kannan et al. [118] deposited 22 layers of super austenitic stainless steel SS904L followed by 22 layers of Hastelloy C-276, and reported a yield strength of 311 MPa, a tensile strength of 681 MPa and a fatigue resistance of 156 MPa at 2 × 10^6^ cycles. Failure initiated in the weaker SS904L region rather than at the interface, so the transition itself was not the limiting feature of this build—one of the few cases in the reviewed evidence where that can be stated from fatigue data.

##### HSLA-Si-Bronze

Marwan et al. [122] produced an FGM by sequentially depositing HSLA steel and silicon bronze (Si-Bronze) by WAAM, and examined the microstructural evolution of the fusion zone between the two dissimilar alloys together with its mechanical properties. Microhardness varied gradually across a 9 mm diffusion zone with no intermetallic compounds detected; fracture occurred in the Si-Bronze region, bending indicated good ductility at the joint and shear strength was comparable to that of HSLA steel. This pairing is therefore one in which a large difference in melting point and thermal expansion did not produce an interfacial weakness, in contrast to the Cu-Al and refractory cases.

##### Ni-Cr-Mo

Singh et al. [123] produced a Ni-Cr-Mo FGM by WAAM and examined the effect of compositional variation and post-deposition friction stir processing on its metallurgical and mechanical properties. The as-deposited samples contained dendritic and columnar grains, whereas the processed samples showed refined, recrystallised grains. Microhardness increased from 147 to 335 HV from the bottom to the top of the samples in both conditions, attributed to CrNi_3_ and MoNi_4_ precipitates, and the tensile strength increased in the same direction.

##### SS316L–Inconel 718

John et al. [124] deposited an SS316L-Inconel 718 FGM using an in-house twin-wire GTAW system operated at 180 A with a travel speed of 3 mm s^−1^ and an interlayer temperature of 150 °C, in which the composition of each layer was governed by the independently controlled feed rates of the two filler wires. A graded block was built from 100% SS316L on one side to 100% Inconel 718 on the other, through intermediate layers of 25%, 50% and 75% Inconel 718, and optical emission spectroscopy confirmed a gradual variation of the alloying elements along the build direction, with the iron content decreasing from 68.4 to 19.9 wt.% and the nickel content increasing from 10.1 to 52.8 wt.%. All four interfaces were metallurgically sound, without cracks, voids or lack of fusion, and X-ray diffraction detected a single austenitic γ matrix throughout the gradient. Microhardness increased monotonically from approximately 210 HV in the SS316L layer to 315 HV in the Inconel 718 layer, with each graded layer exceeding its wrought counterpart, while the intergranular corrosion rate approximately halved in the same direction; tensile properties were not reported. Notably, the layer containing 75% Inconel 718 exhibited hardness and corrosion resistance closely comparable to those of the fully Inconel 718 region. This system reinforces the Ni-superalloy–austenitic-steel pairing already prominent in Table 4, extending it to the widely used precipitation-hardened Inconel 718 alloy.

##### P91 Steel–Inconel 740H

Wang et al. [33] fabricated a P91–Inconel 740H wall in 10 wt.% composition increments by plasma-arc WAAM. Hardness, porosity, composition, microscopy and CALPHAD-derived descriptors were assembled into a high-throughput data set. A genetic algorithm selected descriptors for machine-learning models of hardness and porosity, which were then used to select a 90 wt.% P91 composition. The study is both a further steel–superalloy demonstration and an exception to trial-and-error path selection: the gradient supplied training data for a predictive design step. The authors also reported scale-transfer uncertainty in tensile response and porosity, so the model-guided composition still required build-scale experimental validation.

##### Mg-Al-Si–Mg-Gd-Y-Zn

Han et al. [31] sequentially deposited Mg-Al-Si and Mg-Gd-Y-Zn walls by WAAM. Elemental diffusion produced an approximately 1.4 mm transition in which Al and Si decreased as Gd, Y and Zn increased. Hardness rose from 57 to 90 HV0.5 across the transition; however, the bimetallic tensile strength remained close to that of the Mg-Al-Si constituent, whilst elongation and hardening behaviour approached those of the Mg-Gd-Y-Zn constituent. Fracture occurred in the transition zone. This lightweight system therefore supplies a further example in which different properties do not follow the same interpolation rule.

##### Other Materials

Reisgen et al. [125] produced three two-material combinations by multi-wire WAAM, each following a linear 0–100% transition, and demonstrated that melt-pool composition in C, Mn, Si, S, P, Ni, Cr, Nb and Mo can be set by the wire-feed speeds. The study establishes the actuation principle on which every graded build in Table 4 depends, without characterising the resulting microstructure or mechanical response.

A preliminary high-entropy-alloy route was identified by the other-method search branch. Liu et al. [32] used an arcing-wire/powder hybrid process to grade FeCoCrNiMo_0.2_ high-entropy alloy into ER120S-G steel by continuously changing the wire–powder feed ratio. The study demonstrated spatial control of phase constitution, grain orientation and corrosion response. Because powder was an essential second feedstock, it is treated here as adjacent arc-based evidence rather than as a strict wire-only WAAM system; nevertheless, it shows that the material landscape is beginning to extend beyond conventional Fe/Ni/Cr alloy pairs.

##### Comparative Results Across WAAM-Produced Material Systems

Table 4 contains 24 rows representing 25 material transitions when the Ta-Mo and Mo-W portions of the Ta-Mo-W build are counted separately. P91–Inconel 740H and Mg-Al-Si–Mg-Gd-Y-Zn entered through the documented other-method branch rather than the database pool. The six steel–Inconel rows cite a much larger group of primary reports than the Ta-Mo-W and Ti6Al4V-SS316L transitions, each of which is represented by one report; this imbalance is retained when interpreting apparent compatibility. The high-entropy-alloy example is excluded from the strict wire-only count because powder was its second feedstock. In the tier scheme of Section 2, transitions produced by a programmed change of composition are tier E1; fixed-composition or sequentially deposited bimetallic walls and the wire–powder hybrid build are tier E2. Supplementary Table S3 gives the tier and M1–M5 appraisal of each evidence-bearing study.

Four cross-system observations can be stated at the level of the reported results. First, defect formation depends on the sampled composition path: a dense crack network was reported in the Ta-Mo transition, Fe_2_Ti-associated cracking occurred in the Ti6Al4V-SS316L gradient, and the 10–50 wt.% Inconel 625 interval was mechanically unfavourable in SS308L–Inconel 625, with cracking tendency reported near 20 wt.% Inconel 625 [98,104,111]. In contrast, the sequential SS316L–Inconel 625 structure showed a smooth compositional interface and failed on the stainless-steel side, the SS316L–Inconel 718 gradient had no observable defects in the examined regions, and both SS316L–Inconel 625 gradients studied by Yu et al. were reported as crack-free [105,109,124]. Secondly, the property profiles are not simple linear interpolations between endpoint values: SS308L–Inconel 625 was weakened over 10–50 wt.% Inconel 625; the 25 wt.%-increment SS316L–Inconel 625 build had a local microhardness and tensile-strength minimum at 75 wt.% SS316L; the properties of 75 wt.% Inconel 718 approached those of the fully Inconel 718 layer; and the Mg/Mg bimetal combined strength close to the Mg-Al-Si alloy with elongation and hardening behaviour close to the Mg-Gd-Y-Zn alloy, while fracturing in the transition zone [31,109,111,124]. Thirdly, reported interventions include electromagnetic stirring, targeted in situ heat treatment, post-deposition heat treatment or friction-stir processing, and modification of the composition increment [99,101,109,116,123]. Fourthly, a P91–Inconel 740H gradient was used to train machine-learning models that selected a 90 wt.% P91 alloy; the larger validation build reproduced the predicted mean hardness reasonably well but showed substantial location-dependent porosity and tensile variability [33]. The interpretation and certainty of these observations are developed in Section 4.

#### 3.2.6. Characterisation of Functionally Graded Materials

This subsection bridges the two central research questions by examining characterisation techniques applied to FGMs. Microstructural, compositional, diffraction, indentation and mechanical measurements can resolve important local or averaged responses; the additional challenge addressed here is to combine such evidence with full-field data and an inverse model when a continuous spatial constitutive law is required.

Following the manufacture of FGM, it is essential to characterise the structured material. A variety of techniques have been employed for this purpose, including microstructural analysis, EDX analysis, hardness measurements, fractography, corrosion testing, phase identification, X-ray CT, and mechanical testing. A summary of these methods, used for the characterisation of FGM, is presented in Table 5.

Two of these techniques carry the compositional burden. Microstructural analysis resolves grain size, layer thickness and porosity across a transition, and has revealed zoning differentiated by grain morphology in graded builds [25,105,116,133]. EDX is the usual means of verifying that the designed composition profile was actually achieved in each region, and so underpins domain M2 of the appraisal in Supplementary Table S3 [25,94,98,134].

Three studies in the corpus are particularly informative examples of local chemical or microstructural gradients outside deliberate WAAM. Oxygen-graded titanium was examined by combined local experiments and computational analysis to relate oxygen content to elastic properties [135]; oxidation-induced gradients in Alloy 718 were resolved by local mechanical testing [136]; and a Fe–Cr–C hardfacing deposit was linked from its through-thickness microstructure to quasi-static and dynamic constitutive response [137]. These tier-E3 studies show that local property mapping is feasible when the gradient is measured, but their gradients arise from interstitial uptake, oxidation or hardfacing rather than programmed wire-feed variation. Explosively welded TA10/6061 pipe and TA2/Q235 plate studies further connect interface structure to structural or dynamic-shear performance [138,139]; they are tier-E3 dissimilar-joint analogues, not AM-gradient demonstrations.

The remaining techniques resolve local rather than continuous responses. Hardness mapping tracks the property variation associated with composition, as in TA1/Inconel 625 builds where Inconel-rich regions were harder [121,126,127,128]. Fractography associates fracture-surface morphology with local composition and microstructure [25,116], and corrosion testing shows that resistance follows local composition and microstructure rather than the bulk average [25,95,129,134]. XRD is the standard means of identifying the phases that form along a path, and is the basis of domain M3 [20,23,24,90,91,92,94,95,140]. X-ray CT reconstructs internal defects and porosity non-destructively [130,131,132]. Tensile, compression and bending tests supply the constitutive properties themselves—modulus, Poisson’s ratio, strength and elongation—and have shown these to vary with position along a gradient [20,26,90,95,141].

The diverse material combinations demonstrated by WAAM establish its viability for FGM production. The heterogeneity introduced by compositional gradients and layerwise deposition nevertheless exceeds what conventional testing can resolve. Each technique above returns either a spatially averaged property or a property at a sampled location; none returns a continuous constitutive law along the path, which is the requirement that motivates Section 3.3.

### 3.3. Inverse Mechanical Characterisation

The subsections below trace the progression from classical testing to inverse identification, and examine how far each approach has been demonstrated on gradient-based materials produced by AM.

The identification of constitutive parameters is typically achieved through the implementation of two primary methodologies. On the one hand, mechanical tests are employed to characterise the relevant material properties. These are based on loading and geometry configurations that yield homogeneous and simple kinematic deformation fields. This process allows for closed-form solutions that relate the parameters to the measurements. On the other hand, advances in full-field deformation techniques have established a second approach, which instead exploits heterogeneous stress and strain distributions across the region of interest to achieve material characterisation [142,143]. A key feature of this approach is its high flexibility in the design of new mechanical tests, enabling multiparameter material identification within a single test configuration [144,145,146].

For heterogeneous materials such as FGMs, the limitation is not that conventional tests provide no spatial information. Micro-tensile specimens, indentation maps, local diffraction and tests sampled at several positions can resolve local responses, while equilibrium may produce a nearly uniform axial force even when strain varies with local stiffness. Their limitation is that such local or gauge-averaged measurements do not, by themselves, establish a continuous multiaxial constitutive-parameter field. That objective requires a spatial parameterisation linked to composition or microstructure, heterogeneous kinematic data and an identification procedure whose sensitivity and validation are reported.

#### 3.3.1. Characterisation of Metallic Additive Manufacturing Materials: Classical Approach

The mechanical characterisation of metallic materials produced by AM, notably by DED processes such as WAAM, has historically been conducted using conventional mechanical testing methods. The primary objective of these studies is the manufacturing process itself; therefore, conventional mechanical characterisation is generally performed on homogeneous specimens that are representative of the functional volume of the parts.

Recent research on DED-based AM has increasingly focused on the mechanical, metallurgical, and functional performance of the manufactured parts as summarised in Table 6. The most studied materials include steel alloys (e.g., 316L, ER70S-6 and carbon steel), titanium alloys (e.g., Ti-6Al-4V), aluminium alloys (e.g., Al-5Mg, 5356) and nickel- and cobalt- based superalloys [117,147,148,149]. For that general purpose, the methodologies commonly applied to AM metals include tensile testing, hardness measurements, detailed microstructural analysis via optical and electron microscopy, corrosion testing, impact testing and fractography [117,149,150,151,152]. The types of specimens employed vary from specimens extracted in different orientations (vertical and horizontal), FGM, bimetallic or multilayer specimens, as well as parts with optimised geometries [153,154,155]. A number of studies have also analysed the effects of post-production heat treatment and process parameters, including deposition speed, interlayer cooling time and the use of recycled powders, on the microstructure and mechanical performance of materials [150,156,157]. These scope-mapping studies illustrate the range of reported anisotropy, process-control and heat-treatment questions; they are not used in the cross-study certainty assessment.

The most recent DED and WAAM characterisation studies broaden this scope map. For nickel-based superalloys, a tunable heterostructured design combining M247 and Inconel 718 layers by DED spanned an ultimate tensile strength range of 846–1189 MPa through hetero-interface strengthening, with DIC confirming coordinated deformation between the hard and soft domains [162], whereas aging heat treatment of arc-DED Inconel 625 recovered strength through $\gamma^{\prime \prime }$ precipitation while meeting low-temperature impact requirements [163]. For structural steels, a zig-zag deposition strategy applied to thick-walled low-carbon (ER70S-6) WAAM walls produced nearly isotropic global properties, with fracture toughness governed by local microstructural heterogeneities rather than by the deposition trajectory [164], while DED repair of dissimilar ultra-high-strength steels restored 99% of the base-material tensile strength without post-heat treatment through martensite-variant selection [165]. These contextual examples illustrate the continued use of conventional testing and the emerging complementary use of DIC; they delimit the transition to the evidence-bearing full-field inverse studies examined next.

#### 3.3.2. Characterisation of Metallic AM Materials: Full-Field Approach

Classical tests remain necessary for baseline qualification, but their homogeneity assumption no longer holds once the constitutive parameters vary across the gauge section. Full-field optical techniques, such as DIC, offer an alternative: they measure the heterogeneous fields directly so that one test can yield the complete set of parameters governing a constitutive law [27,166]. The Virtual Fields Method (VFM) extracts them from the measured kinematics through the principle of virtual work, without iterative simulation, whereas Finite Element Model Updating (FEMU) recovers them by updating a finite-element model until its predicted fields match the measured ones. Both are formulated independently of the material system, so their transfer to graded WAAM alloys is a question of evidence rather than of principle.

Experimental applications span welded aluminium and titanium [167,168,169], homogeneous AM metals [170] and architectured AM structures [171]. In LPBF 316L, FEMU-assisted calibration showed that a von Mises description did not reproduce the measured strength-differential effect, motivating a Cazacu–Barlat model [170]. For an LPBF AlSi10Mg lattice, in situ micro-CT, digital volume correlation and force-based FEMU identified parent-strut properties from one compression test; the displacement uncertainty was approximately 0.05 voxel and the recovered strut properties were lower than ex situ dog-bone values, revealing a scale effect [171]. These examples show why heterogeneous fields can be informative but identifiability still depends on the constitutive parameterisation, measurement resolution, boundary conditions and independent validation [143,144]. Recent machine-learning surrogates can accelerate repeated forward calculations [172,173], while physics-based compressive sensing has been proposed to reduce data requirements in AM digital twins [174]; neither approach removes the need to propagate experimental uncertainty or validate recovered parameters.

The wider scope map further spans simultaneous flow-stress/friction calibration, cyclic-damage and dynamic-biaxial identification, thermomechanical and creep tests, physics-informed or invertible neural networks, indentation and imprint inversion, anisotropy calibration, DVC-assisted identification and configurational-force analysis [175,176,177,178,179,180,181,182,183,184,185,186,187,188,189]. Heterogeneous-test design and inverse-strategy studies additionally address Arcan loading, coupled thermal fields, post-necking response, anisotropic plasticity, information richness and sequential identification [190,191,192,193,194,195,196,197,198]. A broader modelling and inversion corpus spans machining, welding, high-temperature tests, ductile damage, impact, composite panels and powder compaction [199,200,201,202,203,204,205,206,207,208,209]. FEMU has also been used to update AM heat-source and thermal-expansion process parameters from measured deformation and temperature fields [210]. This paragraph maps methodological breadth only; these citations do not enter the synthesised claims or their certainty grades. Process-parameter updating or identification in homogeneous specimens does not itself demonstrate recovery of a WAAM spatial constitutive law.

Full-field measurements have supported creep-parameter identification from a notched Inconel 718 specimen, post-necking work-hardening identification in thick high-strength steel, non-linear VFM identification of transformation-based anisotropic plasticity, and hybrid identification of coupled anisotropic plasticity and damage [211,212,213,214]. Cruciform-specimen optimisation and three-dimensional VFM identification further address information content and large-deformation parameter recovery [215,216]; the multi-geometry 304L DIC/thermography data set supplies reusable validation data but is not itself an inverse identification [217]. These studies strengthen the proposed experimental design and uncertainty requirements. They use homogeneous or synthetically heterogeneous specimens, however, and therefore remain method-transfer evidence rather than direct evidence for a spatial law in a WAAM gradient. Avril et al.’s comparison of full-field identification methods is cited for methodological context only and was excluded from the primary corpus as a review article [34].

The decisive boundary for the present review is therefore demonstrational: inverse identification has been performed on homogeneous AM alloys (tier E2) and on incidental gradients in welds (tier E3), but no experimental study within the reviewed Scopus and Web of Science corpus identified a spatial constitutive law across a deliberate WAAM compositional gradient.

#### 3.3.3. Characterisation of Gradient-Based Metallic Materials: Full-Field Approach

This subsection separates three evidential levels that had previously been conflated: experimental inverse identification of incidental gradients in welds (tier E3); full-field measurement, without constitutive inversion, on deliberate AM/WAAM gradients (tier E1); and numerical proof-of-concept on an idealised FGM (tier E4). The numerical literature includes thermal-stress analyses of graded structures and eigenfunction-VFM identification of orthotropic or thermomechanical parameters [218,219,220,221,222]. Table 7 concerns the first and third levels. Its welded joints are useful analogues because their heat-affected zones vary spatially, but their gradients were not designed and their transfer to a compositionally graded build remains a hypothesis.

For welded metals, the VFM has recovered local elastic, yield and hardening behaviour from DIC fields across friction-stir, electron-beam and laser welds [167,168,223,224,225,226]. FEMU has been used for heat-affected-zone gradients and for problems involving a detailed forward model, sparse measurements or damage variables [169,227,228]. Adhesive applications broaden the experimental methodological evidence [229]; the impact-engineering review by Markiewicz et al. [166] is retained only as contextual scope mapping and does not strengthen an alloy-specific conclusion.

Recent weld studies strengthen the analogue evidence without closing the WAAM gap. Koščo and Chmelko identified local constitutive parameters in small S355 weld regions from DIC strains sampled along the notched minimum section and a force-residual objective [230]. Hou et al. combined local indentation, finite-element inversion and DIC validation across superalloy weld micro-zones from 20 to 800 °C, for which a homogeneous-property assumption produced local response deviations of up to 67% in the investigated configuration [231]. Zhu et al. transferred a physics-constrained neural constitutive model between base, weld and heat-affected zones using limited additional data [232]. Each study addresses a naturally produced weld gradient rather than a programmed composition path.

Local or equivalent properties were derived for friction-stirred Al–Cu–Li, high-strength-steel HAZs, welded cold-formed sections, laser-welded DP980 and welded tubes [233,234,235,236,237]. Fracture or deformation localisation was instead measured—without recovery of a continuous constitutive-parameter field—in microalloyed S690QL HAZs, welded steel at low temperature, X80 pipeline welds, joints made by different joining processes, Ti-5111 process zones, local weld-stiffness zones, welded mild steel and fatigued F82H welds [238,239,240,241,242,243,244,245]. The first group strengthens transfer of local calibration and the second strengthens specimen and failure-zone selection; neither group is evidence that VFM or FEMU has identified a spatial law across a deliberate WAAM composition gradient.

**Table 7:** Inverse identification in heterogeneous systems relevant to, but distinct from, deliberate WAAM compositional gradients.

Material	Mechanical Test	Inverse Method	Objective	Tier	Reference
Aluminium welded joint	Tensile test	FEMU	Identification of strength variation in HAZ	E3	[169]
FSW joint—AA6061-T6	Tensile test + DIC + FEM	VFM	Identification of local yield stress	E3	[223]
2024-T3 aluminium (FSW)	Tensile test	VFM	Identification of elastic and plastic properties	E3	[167]
Ti–6Al–4V (EBW)	Tensile test with DIC	VFM	Identification of elastic and plastic properties	E3	[168]
FSW aluminium 2024-T3	Tensile test + DIC	VFM	Identification of local properties	E3	[224]
Composite with FBG sensors	Delamination (monotonic and fatigue)	FEMU	Analysis of delamination using FBG and numerical inversion	E3	[227]
Multi-steel spot weld (DP600 and mild steel)	Not specified	FEMU	Modelling and identification of connector behaviour	E3	[246]
Ductile adhesive	Tensile test at low temperatures	FEMU	Modelling adhesive behaviour under low temperature	E3	[229]
2 mm thick Al-Mg alloy laser-welded	Tensile test + DIC	VFM	Identification of local elasto-plastic properties	E3	[225]
2.5 mm thick aluminium alloy laser-welded	Tensile test + DIC + FEM	VFM	Identification of elasto-plastic parameters with kinematic hardening	E3	[226]
Welded joint (synthetic spatial fields)	Virtual heterogeneous tensile test	Automated VFM	Spatial mapping of heterogeneous elastoplastic properties; synthetic verification only	E4	[247]
Friction-stirred Al–Cu–Li	Tensile test + DIC	Inverse FE identification	Determination of local constitutive behaviour across an incidental process zone	E3	[233]
High-strength-steel HAZ	Local/multiscale tests	FE-supported calibration	Zone-resolved mechanical behaviour in a welded HAZ	E3	[234]
Notched Inconel 718	Creep test + full-field strain	FEMU	Identification of creep parameters in a homogeneous alloy specimen	E3	[211]
Thick high-strength steel	Diffuse-necking test + DIC	FEMU	Post-necking work-hardening identification	E3	[212]
Homogeneous anisotropic sheets	Heterogeneous mechanical tests + DIC	Non-linear VFM	Identification strategy for transformation-based anisotropic plasticity	E3	[213]
Cruciform specimen (synthetic fields)	Biaxial test design	Integrated DIC/FEMU	Optimisation of geometry for constitutive-parameter identifiability	E4	[215]
Homogeneous metal (synthetic 3-D fields)	Large-deformation test	VFM	Plastic-parameter identification from three-dimensional displacement fields	E4	[216]
FSW aluminium alloy (Al-Cu)	Simulated tensile test	FEMU	Simulation of damage and fracture using GTN inverse fitting	E4	[228]

DIC: Digital Image Correlation; EBW: Electron BeamWelding; FBG: Fibre Bragg Grating; FEM: Finite Element Method; FEMU: Finite Element Model Updating; FSW: Friction Stir Welded; HAZ: Heat-Affected Zone; VFM: Virtual Fields Method.

For an intentional FGM, Nayakanti and Subramanian recovered a spatially varying elastic modulus with an eigenfunction VFM [248], but the strain fields were generated numerically. This is a method verification, not an experiment on a manufactured gradient. A corresponding FEMU analysis would additionally require a forward model and a sufficiently low-dimensional description of the spatial parameter field.

Full-field measurement has now reached deliberately graded builds. In a T-WAAM Inconel 625–SS308L interlayer, DIC accompanied refined grains and a 41% reduction in oxidation ingress relative to the direct joint [249]. In SS316L–Cu CMT-WAAM, DIC located fracture initiation at a surface fissure near the interface, whose thermal conductivity was approximately 164 W m^−1^ K^−1^ [250]. For an AM 316L–Inconel 718 interface, a deliberately positioned notch redirected failure and increased strength and toughness by 40% and 20% at room temperature, with larger gains at 650 °C [251]. A further composition-gradient specimen combined high-resolution DIC with data-driven mapping to estimate fatigue strength from one graded coupon [252]. These studies provide experimental platforms and spatial measurements, but they report measured fields or derived local indicators rather than a constitutive-parameter field identified by VFM or FEMU.

Adjacent heterogeneous-material studies were retained only to define transfer limits. DIC has resolved deformation in a graded LPBF lattice, a single-material WA-DED Grade 91 build and an architectured ferritic steel, while a bioinspired polymer system demonstrates geometric and material grading outside the metallic scope [253,254,255,256]. These studies can inform specimen design or field interpretation, but they do not add experimental evidence for constitutive inversion across a deliberate metal–metal WAAM composition path.

##### Demonstrated Capability and Proposed Transfer

The evidence supports a narrower conclusion than a direct allocation rule. Deliberate WAAM/AM composition gradients have been measured by DIC at tier E1 [249,250,251,252], while spatial identification by VFM or FEMU has been demonstrated on incidental weld gradients at tier E3 [167,168,169,233,234]; the FGM-specific eigenfunction VFM demonstration and the automated welded-property map used synthetic finite-element fields and are tier E4 [247,248]. These two capabilities have not been joined: no experimental study within the reviewed Scopus and Web of Science corpus identified a spatial constitutive law across a deliberate WAAM compositional gradient. Combining them is accordingly proposed here as a research priority, and the workflow set out in Section 4 is a hypothesis to be tested rather than a validated WAAM methodology.

The often-stated preference for VFM with dense kinematic fields and for FEMU with complex forward models is therefore retained only as a scenario-derived starting hypothesis [27,34,166]. The reviewed dual-database corpus contains no same-specimen, same-constitutive-model comparison on a deliberate gradient. A defensible method choice must consequently be made after comparing parameter sensitivity, identifiability, noise propagation, computational cost and held-out prediction for the intended specimen and constitutive law.

## 4. Discussion

The central result is not a universal ranking of alloy pairs, but a sequence for testing compatibility claims. The endpoints of a gradient may each be weldable while an intermediate mixture enters a brittle phase field, changes solidification mode or develops a larger thermal-strain incompatibility. Dilution and repeated reheating can then shift the realised path away from the programmed wire-feed ratio. Process optimisation can reduce lack of fusion, porosity and heat accumulation; it cannot by itself remove an intrinsically unfavourable phase field. Table 8 sets out the claim-level evidence assessment on which the following discussion rests.

The larger steel–superalloy sample creates a clear selection effect. The mapped evidence contains substantially more primary reports for the six steel–Inconel transitions than for refractory, titanium and magnesium combinations, which are often represented by a single system or report. Their apparent success must therefore not be interpreted as an intrinsic alloy-family advantage or generalised to all arc sources, transfer modes and thermal histories. The evidence supports a candidate window for fusion-based GTAW/GMAW/plasma deposition, not universal compatibility. Conversely, a crack in a sparsely studied pair does not prove that every gradient path is infeasible.

A practical decision sequence follows from this qualification. First, calculate or measure equilibrium and non-equilibrium phase stability along the entire proposed composition path. Secondly, examine solidification range, dilution, segregation and thermal-expansion mismatch together because no single descriptor is sufficient. Thirdly, fabricate short graded coupons that traverse the predicted critical windows and verify actual composition, phases, porosity and cracking before committing to a component-scale build. Only then should wire-feed ratio, heat input, interpass temperature, deposition sequence and post-treatment be optimised. Mechanical validation must sample both end members and every critical intermediate window.

This sequence is an evidence-informed proposal, not a validated decision map, and its four stages do not rest on evidence of equal strength. Stage 3—that a critical interval must be fabricated and inspected before a component-scale build—is the best supported: intermediate-window failure independent of endpoint quality is reported in the Ta-Mo, Ti6Al4V-SS316L and SS308L–Inconel 625 systems, and locally anomalous properties in intervals that did not crack are reported in three further transitions [31,98,104,109,111,124]. Stage 4—that process variables should be optimised only after the material path has been checked—is supported at the level of mechanism by studies in which process interventions improved global properties without removing the local transition risk, most directly the hybrid LMD–WAAM route that raised tensile strength by 8% while moving fracture to an internal composition interface [99,101,114]. Stages 1 and 2, by contrast, are extrapolated. No study in the reviewed evidence prospectively screened a composition path, predicted a critical interval and then confirmed that prediction experimentally on a WAAM build; the closest case is the P91–Inconel 740H workflow, in which CALPHAD descriptors and machine-learning models selected a composition that still required a confirmation build [33]. The first two stages should therefore be read as a rational ordering of existing screening tools, inferred from a small number of alloy families, rather than as a procedure whose predictive value has been demonstrated.

Thermodynamic screening alone is particularly insufficient wherever the realised path departs from the nominal one or the relevant phases are not the equilibrium ones. Four such conditions recur in the reviewed evidence. Non-equilibrium solidification under the cooling rates typical of arc deposition can suppress predicted phases or retain metastable ones, as in the Mo-W transition, where composition and hardness varied sharply through the first layers because of the melting-point difference and rapid solidification of the melt pool [98]. Dilution moves the local composition away from the programmed wire-feed ratio, so the interval actually traversed may not be the interval screened; remelting between layers smooths a nominally stepwise path into a different continuous one. Segregation redistributes solute within the melt pool, so a composition that is acceptable on average may be locally unfavourable. Repeated thermal cycling then re-ages the deposited material, which is why an interval that is sound as deposited may still develop phases on subsequent passes, and why interpass temperature changes the resulting microstructure in the Ti-Al system [93,94]. Equilibrium calculation is accordingly a screening filter that identifies intervals warranting experimental attention; it does not certify an interval as safe, and a negative screening result is more reliable than a positive one.

Transfer of the sequence between alloy families is likewise limited, and the limits differ by system. For Fe-based and Ni-based pairs, the sequence is best supported, because the intermediate compositions remain largely austenitic and the dominant risks—local weakening, segregation and window-specific cracking—are those the sequence is designed to detect. For Ti-based pairs the governing risk is the formation of brittle intermetallics such as Fe_2_Ti over narrow intervals [104], so the resolution of the coupon in stage 3 matters more than in the steel–superalloy case, and a coarse composition increment may step over the critical window entirely. For Cu-based pairs the difficulty is the combination of intermetallic formation with a large difference in thermal conductivity and expansion [24,26,250], which makes the thermal terms in stage 2, rather than phase stability alone, the discriminating factor. For refractory pairs the evidence reduces to one build [98], and the wide melting-point separation means that composition and thermal history vary steeply over very few layers, so neither the ordering nor the coupon length proposed here has been tested at an appropriate scale. For Mg-based and high-entropy-alloy systems, each represented by a single recent study [31,32], the sequence is untested. Applying it outside the Fe-Ni evidence base is therefore itself a hypothesis, and the framework should be reported with the alloy family, arc process and composition interval for which it was exercised.

The recent P91–Inconel 740H study adds a useful design layer: CALPHAD-informed experiments, a genetic algorithm and machine-learning regressors were combined to select a gradient from a finite WAAM data-set [33]. Such models can prioritise experiments and expose interactions that a linear rule misses. Their predictions nevertheless inherit the composition range, process window and measurement uncertainty of the training builds. Reporting cross-validation alone is insufficient; uncertainty bounds, extrapolation limits and confirmation builds at the selected composition are required before a machine-designed path can be described as compatible.

### 4.1. Operational Validation of a Spatial Constitutive Law

Table 9 specifies the benchmark experiment that would test the central hypothesis of this review. It is a proposed protocol, not a validated one: the complete sequence has not been executed on a deliberate WAAM compositional gradient within the reviewed Scopus and Web of Science corpus, and the evidential-basis column records the tier from which each specification is transferred. The paragraphs that follow give the reasoning behind the principal choices.

A first experimental benchmark should machine a dog-bone from a graded wall so that the gauge length spans the full composition path in the loading direction; a transverse specimen and a separately manufactured wall should provide held-out tests. The realised composition, thickness and phase field should be registered to the specimen before loading. Stereoscopic or matched front–back DIC, synchronised with force, is preferable where wall curvature or bending is possible. The report should state subset size, spatial resolution, strain uncertainty and the number of independent pixels across each transition.

Quasi-static monotonic tension with at least one unload–reload segment can identify elasticity, yield and hardening; a notched or off-axis geometry is required if the target model includes multiaxiality or damage. Spatial parameters should be represented by a low-dimensional, physically constrained basis—for example, piecewise-linear or spline functions tied to measured composition—rather than by an independent parameter at every pixel. A VFM implementation would construct virtual fields selected for sensitivity to these coefficients; a FEMU implementation would minimise a weighted objective combining force and displacement fields. In both cases, parameter correlation, regularisation choice, image noise and boundary-condition uncertainty must be reported. Recovery from synthetic fields is only a verification step. Validation requires prediction of withheld load increments, local hardness or micro-tensile measurements, and preferably a second specimen or loading direction.

This workflow is deliberately method-neutral. Dense surface fields and a modest elasto-plastic parameterisation make VFM an efficient candidate; complex geometry, three-dimensional fields or internal-variable damage models may favour FEMU. These are testable expectations, not conclusions established by the present evidence. A head-to-head comparison using identical data, parameterisation and validation metrics is needed before either method can be recommended for WAAM gradients.

### 4.2. Review Limitations

Searching two multidisciplinary databases does not make the search exhaustive. The subject spans Additive Manufacturing, welding metallurgy, materials science, experimental mechanics and inverse identification, which are indexed unevenly across multidisciplinary and specialist engineering databases. The queries were also built around Functionally Graded Materials, WAAM and inverse identification, so the corpus over-represents WAAM relative to the wider additive-manufacturing literature; what it contains describes the scope of this review and not the distribution of work in the field. Scopus and Web of Science also search different fields: the Web of Science Topic field includes Keywords Plus and is not identical to Scopus TITLE-ABS-KEY. Web of Science query v was not run separately because query vi is its strict superset, and no interface screenshots survive; the raw exports, syntax and checksums nevertheless preserve the retrievable search evidence. The other-method branch yielded three eligible reports outside the 882-record database pool, confirming that database indexing alone is not saturated. Statements of non-demonstration are therefore bounded to the assembled dual-database corpus and would be overturned by one qualifying study outside it.

The synthesis is also constrained by its English-language eligibility, uneven terminology and strongly unequal study numbers between alloy families. Work described as wire-arc DED rather than WAAM may have been missed on terminology alone. Heterogeneous compositions, process variants and outcome definitions precluded meta-analysis. Publication bias towards successful builds is also plausible and cannot be tested here because the small and uneven number of studies per material system makes the usual asymmetry diagnostics uninformative. The compatibility map and Table 8 should consequently be read as an evidence-weighted decision aid, not as a certified database of material pairs.

## 5. Conclusions

The conclusions are separated by evidential status. Each statement below carries the certainty grade recorded in Table 8, using the review-specific scheme defined in Section 2; those grades are an organisational device for this corpus and are not GRADE or risk-of-bias ratings. No conclusion in the first group is graded high, and none should be read as applying beyond the alloy families, arc processes and composition intervals named in the cited builds.

- Established within the reviewed: Scopus and Web of Science corpus. Deliberate composition gradients have been fabricated by several WAAM variants, but their literature is unevenly distributed towards steel–Ni-superalloy systems. Sound endpoints do not guarantee a sound transition: phase formation, dilution, solidification and thermal strain act along the complete composition path, an inference drawn from three tier-E1 systems and graded moderate. Steel–Inconel paths have produced metallurgically sound transitions within the composition intervals validated in the cited builds (moderate); this is not a statement of universal compatibility for the family, because cracking is also reported in specific intermediate windows of the same alloy pairs, and the family’s apparent advantage partly reflects its much larger share of the evidence. Departures from simple endpoint interpolation are reported in at least four distinct transition systems (moderate), although the affected property differs between them. Cracking associated with intermetallic formation on Ti–Fe/Ni paths is reported consistently in direction across three systems (moderate), and the contribution of thermal-expansion mismatch to refractory-pair cracking rests on one comparative build and is low/preliminary; neither finding should be generalised to other alloy families. Full-field measurement has reached deliberately graded AM builds, but these studies report measured fields rather than identified parameters: no experimental study within the reviewed dual-database corpus identified a spatial constitutive law across a deliberate WAAM compositional gradient by VFM or FEMU. That bounded negative result remains low/preliminary because differences in database fields, terminology and unsearched specialist sources prevent a claim of universal absence; one qualifying study would overturn it.
- Evidence-based recommendations: Candidate pairs should be screened along the whole composition path before process optimisation, and predicted critical windows should be checked on short coupons by compositional, phase, defect and mechanical measurements. The strength of this recommendation is uneven: the requirement to fabricate and inspect a critical interval before a component-scale build is supported across several tier-E1 systems, whereas the prior screening steps are extrapolated from a small number of alloy families and have not been prospectively validated on a WAAM build. Outside Fe- and Ni-based pairs—and particularly for Cu-based, refractory, Mg-based and high-entropy systems, each represented by one or two studies—the sequence is itself untested and should be applied as a hypothesis. Claims of compatibility should name the arc process, transfer mode, thermal history and validated composition interval. Machine-learning models can guide this sequence only when their uncertainty, domain of applicability and confirmation builds are reported.
- Testable hypotheses and priorities: Nothing in this group is an established result. A specimen whose gauge length spans a measured WAAM gradient may permit a low-dimensional spatial constitutive law to be identified from one heterogeneous test; the protocol in Table 9 is proposed as the experiment that would test this, and no element of it has yet been executed on a deliberate WAAM gradient. VFM may be efficient for dense fields and modest elasto-plastic models, whereas FEMU may be advantageous for complex geometries or internal-variable models; this proposed division is a scenario-derived heuristic rather than a finding and requires a controlled, same-data comparison. Less-studied titanium, aluminium, magnesium, refractory and high-entropy-alloy transitions require replicated studies before their performance can be compared fairly with the steel–superalloy evidence base.

## Figures and Tables

**Table 1:** Shared search bodies for the dual-database update covering eligible publications up to and including 31 July 2026. Database-specific field wrappers, calendar-year bounds and query-specific raw occurrence counts are reported in Supplementary Table S2. Web of Science query v was not separately executed because query vi is its strict superset.

No.	Query Body
i	“functionally graded material” AND “mechanical test”
ii	“functionally graded materials” AND “wire and arc additive manufacturing”
iii	“additive manufacturing” AND (“virtual fields method” OR “inverse identification” OR “FEMU” OR “material testing 2.0”)
iv	(“DED” OR “wire and arc additive manufacturing”) AND “mechanical test” AND “mechanical properties”
v	“functionally graded material” AND (“virtual fields method” OR “inverse identification” OR “FEMU” OR “material testing 2.0”)
vi	“functionally graded material” AND (“virtual fields method” OR “inverse identification” OR “FEMU” OR “material testing 2.0” OR “digital image correlation”)
vii	“alloys” AND (“virtual fields method” OR “inverse identification” OR “FEMU” OR “material testing 2.0”)
viii	“metal” AND (“virtual fields method” OR “inverse identification” OR “FEMU” OR “material testing 2.0”)
ix	(“welded” OR “functionally graded material”) AND (“virtual fields method” OR “inverse identification” OR “FEMU” OR “material testing 2.0” OR “digital image correlation”)

**Table 2:** Classification of FGM types according to gradient characteristics.

FGM Type	Description	Examples	Typical Applications
Chemical composition gradient	Progressive alteration of chemical composition within the bulk material; can be single or multiphase	Metal-ceramic (e.g., Ti/HAP, ZrO_2_/Ni), Metal-metal (e.g., Fe-Al, Ti-Al, Ni-Ti)	Thermal barrier coatings, biomedical implants, aerospace components
Porosity gradient	Spatial variation in pore density or pore size across the material volume	Graded density foams, porous scaffolds	Biomedical scaffolds, lightweight structures, filtration systems
Microstructure gradient	Gradual alteration of microstructure through controlled processing	Heat-treated steels, surface-hardened components	Wear-resistant surfaces, fatigue-resistant components

**Table 3:** Summary of methods for manufacturing FGMs.

Category	Methods	References
Gas based methods	Chemical vapour deposition	[40]
	Physical vapour deposition	[41]
	Thermal spray	[42]
	Surface reaction process	[43]
Liquid phase methods	Centrifugal casting	[44,45]
	Combustion	[46]
	Tape casting	[47]
	Slip casting	[48]
	Gel-casting	[49]
	Electrophoretic deposition	[50]
	Chemical solution deposition	[49]
	Laser deposition	[51,52]
	Directional solidification	[53]
	Sedimentation	[54]
	Electrochemical gradation	[55]
Solid phase methods	Spark plasma sintering	[56]
	Powder metallurgy	[57,58]

**Table 4:** Summary of composition-transition and graded material systems produced by Wire-fed Arc Additive Manufacturing. The table distinguishes programmed gradients (tier E1) from fixed-composition or sequential bimetallic arc deposits (tier E2) in the text and Supplementary Table S3; inclusion in the table is not itself evidence of a continuous gradient.

Materials	Functional Gradient	Examples of Industrial Application	Refs.
Fe-Al	Al gradient 15 to 55 at (%)	Applications requiring high-damage resistance	[20,90]
Fe-Ni	20 to 50% of Ni	Aeronautical and maritime industries	[21,91,92]
Ti-Al	20 to 50% of Al	Aerospace and automotive	[22,93,94]
Ni-Ti	50% of Ni and 50% of Ti	Automotive, aerospace, robotic, and biomedical	[23,95,96]
Cu-Al	99.9% Cu wire and 1080-grade Al wire, both 0.9 mm in diameter; also produced as a graded library of Cu-CuAl compositions	Shipbuilding industries	[24,97]
Ta-Mo-W	100% Ta 0% Mo to 0% Ta 100% Mo to 0% Mo 100% W	High-temperature applications	[98]
HSLA-Cu-Al	25 layers of HSLA steel and 20 layers of Cu-Al alloy	Applications that require high thermal/electrical conductivity, wear resistance with excellent mechanical properties	[26]
Inconel 625–HSLA	100% Inconel 625 0% HSLA to 0% Inconel 625 100% HSLA	Demanding environments such as pressurised water reactor systems and pipe-to-safe-end connections	[99,100,101,102]
Ti6Al4V–Inconel 625	100% Ti6Al4V 0% Inconel 625 to 0% Ti6Al4V 100% Inconel 625	Applications in extreme environments, including aerospace engineering, nuclear power generation, sensors, biomedical implants, optoelectronic devices, and absorption systems	[103]
Ti6Al4V-SS316L	5at.% Fe to 65at.%Fe	Applications that require excellent corrosion resistance, high strength-to-weight ratio and low cost	[104]
Inconel 625–SS316L	Sequential SS316L/Inconel 625 deposits and layerwise gradients with 25 wt.% or 100 wt.% composition changes	Aerospace, power generation, petrochemical, chemical and marine applications	[105,106,107,108,109]
SS308L–Inconel 625	100% SS308L to 100% Inconel 625, graded either along the build direction or along the torch travel direction	-	[110,111,112,113]
Inconel 625–SS304L	75% SS304L 25% Inconel 625 to 25% SS304L 75% IN625	-	[114]
DSS-CMS	Half of the wall is duplex stainless steel and the other half is carbon manganese steel	Marine risers	[25,115]
Low-carbon steel–SS316L	Half of the wall is low-carbon steel and the other half is SS316L	Bimetallic structures	[116]
LAS-ASS	Layers of each material deposited interspersed	-	[117]
SS904L–Hastelloy C-276	22 layers of SS904L followed by 22 layers of Hastelloy C-276	Harsh environments such as oil and gas industries and nuclear power plants for manufacturing steam generator, reformers	[118]
Inconel 825–SS316L	20 layers of Inconel 825 and 20 layers of SS316L	Environments that require corrosion-resistant alloys	[119,120]
TA1–Inconel 625	100% TA1 and 0% Inconel 625 to 100% Inconel 625 and 0% TA1	Extreme environment conditions	[121]
HSLA-Si-Bronze	100% HSLA and 0% Si-Bronze to 100% Si-Bronze and 0% HSLA	-	[122]
Ni-Cr-Mo	10 layers with different % Ni, Cr and Mo	Defense, aerospace, and nuclear applications	[123]
SS316L–Inconel 718	Five layers from 100% SS316L to 100% Inconel 718 (25%, 50% and 75% intermediate), set by independently controlled twin-wire feed rates	Corrosion-resistant components for pressure vessels and piping in power-plant, nuclear and petrochemical service	[124]
P91 steel–Inconel 740H	Wall graded from P91 steel to Inconel 740H in 10 wt.% increments; a 90 wt.% P91 composition was subsequently selected by a machine-learning workflow	High-temperature components requiring spatial control of hardness and porosity	[33]
Mg-Al-Si–Mg-Gd-Y-Zn	Sequentially deposited Mg-alloy walls with an approximately 1.4 mm diffusion-controlled transition zone	Lightweight aerospace and automotive structures	[31]

HSLA: High Strength Low Alloy; SS316L: Stainless-steel 316L; 308L: Stainless steel 308L; 304L: Stainless steel 304L; DSS: Duplex stainless steel; CMS: Carbon-manganese steel; LAS: Low alloy steel; ASS: Austenitic stainless steel; SS904L: Super austenitic stainless steel; TA1: commercially pure titanium (Grade 1).

**Table 5:** Summary of characterisation techniques for FGMs.

Technique	Purpose	Parameters Analysed	References
Microstructure	Internal structure analysis	Grain size, coating thickness, porosity	[25,105,116]
EDX	Elemental analysis	Chemical composition, present elements	[25,94,98]
Hardness	Resistance to plastic deformation	Brinell, Vickers, Rockwell hardness	[126,127,128]
Fractography	Fracture surface analysis	Failure modes, crack propagation	[25,116]
Corrosion Testing	Degradation assessment	Performance in corrosive environments	[25,95,129]
Phase Identification (XRD)	Crystalline phase analysis	Phase identification, crystalline composition	[20,23,24,90,91,92,94,95]
X-ray CT	Non-destructive 3D imaging	Internal structure, phase distribution	[130,131,132]
Mechanical Testing	Mechanical properties evaluation	Strength, ductility, Young’s modulus, Poisson’s ratio	[20,26,90,95]

EDX: Energy-dispersive X-ray; X-ray CT: X-ray Computed Tomography.

**Table 6:** Summary of materials, processes, tests, and objectives reported in the characterisation of metallic AM using DED processes.

Material	Process	Specimen Type	Methods	Objective	References
Dissimilar steels, bronze, bimetallic	WAAM	Multilayer and bimetallic specimens	Tensile test, hardness, microstructure	Analysis of interfaces, functional gradients and transition zones	[117,148,158]
Structural steels, carbon steels	WAAM	Vertical and oriented test specimens	Tensile test, hardness, microstructure	Optimisation of parameters and effects of heat treatment	[150,153,154]
Titanium and Ti-Nb alloys	DED (Laser)	Multilayer and gradient samples	Tensile test, hardness, microstructure	Influence of recycling, functional gradients and interfaces	[149,155,157]
Ni and Co–Cr superalloys	Hot-wire TIG and DED	Post-treated specimens	Tensile test, hardness, fractography	Optimisation of post-heat treatment and mechanical evaluation	[151,159]
Aluminium alloys, bronze alloys	WAAM	Oriented and multilayer specimens	Tensile test, hardness, fractography	Study of anisotropy, porosity and multidirectional properties	[152,156,160]
316 stainless steel with tungsten	DED (Laser)	Modified surface layers	Tensile test, corrosion, microstructure	Improvement in corrosion resistance and mechanical properties	[161]

**Table 8:** Claim-level evidence assessment. “Certainty” is a review-specific organisational grade defined in Section 2; it concerns the stated cross-study claim, not the quality of every paper in the group, and it is not equivalent to GRADE or to a formal risk-of-bias rating. “Tier” and the appraisal domains are those defined in Section 2 and applied study by study in Supplementary Tables S3 and S4.

Claim	Evidence Base and Tier	Consistency and Governing Appraisal Limitation	Rule Applied	Certainty
Steel–Inconel paths can produce sound transitions within the validated composition intervals of the cited builds	Primary reports across six mapped steel–Inconel transitions, including database and other-method evidence; tiers E1–E2, the E2 entries being fixed-composition or sequentially deposited builds [33,99,105,106,110,114,119,124]	Several sound builds, but cracking also occurs in particular intermediate windows, which the reports attribute to identified intervals rather than to unexplained scatter; the family is studied much more often than alternatives. M4 only partly met across the group: replicate counts and scatter are seldom reported and one system reports no tensile data	Provisional high; appraisal downgrade on M4; contradiction retained as a stated boundary	Moderate
Intermetallic-forming Ti–Fe/Ni paths carry a high cracking risk	Three reported systems, each represented by a small number of studies; tier E1 [103,104,121]	Phase formation and cracking are directionally consistent, but process variants and replication are limited. M3 met; M4 partly met, with mechanical evidence confined to selected locations	Provisional high on system count; appraisal downgrade on M4	Moderate
Thermal-expansion mismatch contributes to refractory-pair cracking	Within-study contrast between Ta-Mo and Mo-W; tier E1, one system [98]	Mechanistically plausible and internally consistent, but based on one comparative system. M3 met; the design does not isolate thermal expansion from phase and process effects, so M5 is partly met	Provisional low/preliminary on replication; no upgrade available	Low/preliminary
Composition–property responses can depart from simple endpoint interpolation	SS308L–IN625, SS316L–IN625, SS316L–IN718 and Mg-alloy transitions; tiers E1–E2 [31,109,111,124]	Local departures are reported across four systems, but the affected property and the mechanism differ between them. M2 met throughout; M4 partly met, and the differing outcome measures prevent a common effect size	Provisional high; appraisal downgrade on M4	Moderate
No experimental study within the reviewed Scopus and Web of Science corpus identified a spatial constitutive law across a deliberate WAAM compositional gradient	Deliberate-gradient full-field measurements (tier E1), experimental weld/local-property identification (tier E3), and numerical spatial-identification demonstrations (tier E4) [167,169,233,234,247,248,249,250,252]	The closest full texts consistently separate measurement on deliberate gradients from inverse identification on incidental weld gradients. I5 is not met in any tier-E1 study because none performs an identification to validate. Dual-database coverage reduces, but cannot eliminate, the risk of a missed study; one qualifying report would overturn the negative claim	Bounded to the reviewed corpus; directness downgrade for reliance on E3–E4	Low/preliminary
The proposed VFM/FEMU allocation is a scenario-derived heuristic, not a validated selection rule	Indirect comparison of unlike specimens and constitutive targets; tiers E2–E4 [170,171,226,247]	No head-to-head assessment on a deliberate WAAM gradient. I4 and I5 cannot be assessed across studies because the compared works use different specimens, models and validation targets	Directness downgrade; no tier-E1 evidence	Low/heuristic

**Table 9:** Proposed operational workflow for identifying a spatial constitutive law across a deliberate WAAM compositional gradient. The workflow is a testable research protocol; the evidential basis column gives the tier (Section 2) of the evidence from which each specification is transferred, and no row is supported by a completed tier-E1 identification.

Element	Proposed Specification	Rationale	Basis
Gradient specimen	Flat dog-bone machined from a graded wall with the gradient direction parallel to the loading axis; grip regions in end-member material only. A transverse specimen and a specimen from an independently manufactured wall serve as held-out tests	One coupon must contain every composition state whose parameters are to be identified; the two held-out specimens separate spatial parameter variation from build-to-build variability	E1, E3
Coverage of the composition path	Gauge length set from a measured composition profile of the parent wall so that both end members and every predicted critical interval lie inside it; composition re-scanned on the machined specimen face rather than on a sibling coupon	Dilution and remelting displace the realised path from the programmed wire-feed ratio, so the interval actually traversed must be measured on the tested surface	E1
Loading mode	Quasi-static monotonic tension with at least one unload–reload segment; a notched or off-axis geometry added when the target model includes multiaxiality or damage	With the gradient parallel to the load the axial force is nearly uniform while strain varies with local stiffness, so one test samples one stress level per composition; the unload–reload separates elastic modulus from plastic slope; uniaxial tension cannot excite the stress states a yield surface or damage law requires	E3
DIC dimensionality and resolution	Stereoscopic DIC, or matched front–back two-dimensional systems, synchronised with force. Subset size and step chosen to give several independent subsets across the narrowest interval to be resolved; subset, step, virtual strain-gauge length, strain resolution and noise floor all reported	The spatial resolution of the identified parameter field cannot exceed the virtual strain-gauge length, so it must be fixed by the narrowest composition feature; wall curvature and bending bias planar DIC through out-of-plane motion	E2, E3
Registration with composition and microstructure	Fiducial marks machined before speckling; composition and phase or hardness maps acquired on the tested face in the same frame; affine registration to the DIC coordinate system with the residual reported	The parameterisation ties parameters to composition, so registration error propagates directly into parameter error and must be quantified rather than assumed negligible	E1
Spatial parameterisation	Parameters expressed as smooth low-dimensional functions of the registered composition coordinate—piecewise-linear or spline with few control points—with positivity or monotonicity constraints where physically required; model order chosen by a stated selection criterion	An independent parameter at every pixel is not identifiable from one test; the low-dimensional basis is the primary regularisation and makes the recovered field interpretable against composition	E4
Inverse method and regularisation	VFM with virtual fields designed for sensitivity to the retained coefficients, or FEMU minimising a weighted force-and-displacement objective. Any penalty term beyond the basis reported with its weight and the criterion used to select it	Both formulations are material-system independent; the choice is an open question for gradients, so the method, its regularisation and its selection criterion must be reported rather than assumed	E3, E4
Identifiability and uncertainty	Objective sensitivity to each retained coefficient; parameter covariance and correlation; conditioning of the sensitivity matrix; propagation of image noise through synthetic images; sensitivity to boundary-condition and registration uncertainty. Non-identifiable coefficients fixed or removed, not reported as identified	Reporting a parameter field without its conditioning gives no basis for judging which coefficients the experiment actually constrained	E2, E3
Independent validation	Prediction of withheld load increments; comparison against local measurements not used in the identification, such as micro-tensile coupons or hardness at selected compositions; repetition on the transverse specimen and the independently manufactured wall	Recovery from synthetic fields verifies the solver, not the constitutive description; validation requires data the identification did not see	E2

## Data Availability

The systematic review protocol is registered in the Open Science Framework (OSF; DOI: https://doi.org/10.17605/OSF.IO/E3TGV). The study-level accounting, database-specific search counts and study-level appraisal results are provided as Supplementary Tables S1–S4 accompanying this article. The raw database exports for Scopus and Web of Science Core Collection, their SHA-256 checksums, the record-occurrence and deduplication files, and the screening and full-text decision files are retained by the authors and are available from the corresponding author upon reasonable request.

## Supplementary Materials

The following supporting information can be downloaded at: https://www.mdpi.com/article/10.3390/ma19163379/s1. File S1: PRISMA 2020 Checklist, recording the location at which each checklist item is reported in the manuscript and in the Supplementary Materials; Table S1: Study-level accounting for every included study; Table S2: Query-by-query database result counts; Table S3: Study-level appraisal of the manufacturing evidence against domains M1–M5; Table S4: Study-level appraisal of the inverse-identification and full-field evidence against domains I1–I5. Table S1 records whether each cited work is an included study, its role, the applicable appraisal framework, and either the supplementary appraisal table in which its ratings appear or a coded reason why the claim-specific domains are not applicable.

## Abbreviations

The following abbreviations are used in this manuscript:

AM	Additive Manufacturing
BAMS	Bimetallic Additively Manufactured Structures
DED	Directed Energy Deposition
DIC	Digital Image Correlation
DLD	Direct Laser Deposition
DMLS	Direct Metal Laser Sintering
DMD	Direct Metal Deposition
EBW	Electron Beam Welding
EDX	Energy-dispersive X-ray
EMS	Electromagnetic Stirring
FBG	Fibre Bragg Grating
FEA	Finite Element Analysis
FEM	Finite Element Method
FEMU	Finite Element Model Updating
FGM	Functionally Graded Material
FGMs	Functionally Graded Materials
FGS	Functionally Graded Scaffolds
FSW	Friction Stir Welded
GTAW	Gas Tungsten Arc Welding
GTN	Gurson-Tvergaard-Needleman
HAZ	Heat-Affected Zone
HSLA	High Strength Low Alloy
IHT	In situ Heat Treatment
LCS	Low Carbon Steel
LDMD	Laser Direct Metal Deposition
LENS	Laser Engineered Net Shaping
LMD	Laser Metal Deposition
LPBF	Laser Powder Bed Fusion
MT 2.0	Material Testing 2.0
OTS	Optimised Transition Strategy
PBF	Powder Bed Fusion
PRISMA	Preferred Reporting Items for Systematic Reviews and Meta-Analyses
SEM	Scanning Electron Microscope
SLM	Selective Laser Melting
SLS	Selective Laser Sintering
TIG	Tungsten Inert Gas
T-WAAM	Twin-Wire Arc Additive Manufacturing
VFM	Virtual Fields Method
WAAM	Wire Arc Additive Manufacturing
X-ray CT	X-ray Computed Tomography
XRD	X-Ray Diffractometer
