# Supplementary material for: Functionally Graded Materials by Wire Arc Additive Manufacturing: Material-Pair Compatibility and Spatial Mechanical Characterisation—A Systematic Review
Source: Materials (Basel). 2026 Aug 8;19(16):3379. doi: 10.3390/ma19163379 (PMC13514652; doi:10.3390/ma19163379)
Supplement: Supplementary file 1 [file materials-19-03379-s001.zip › materials-4466069_PRISMA_2020_checklist.pdf]

PRISMA 2020 Checklist

Manuscript ID: materials-4466069

Title: Functionally Graded Materials by Wire Arc Additive Manufacturing: Material-Pair Compatibility and Spatial Mechanical Characterisation—A Systematic Review

Page and line references correspond to the final revised manuscript PDF (43 pages). Supplementary-page references correspond to the final 16-page Supplementary Materials PDF containing Tables S1–S4.

Section and Topic	Item #	Checklist item	Location where item is reported
**TITLE**			
Title	1	Identify the report as a systematic review.	Title, p. 1.
**ABSTRACT**			
Abstract	2	See the PRISMA 2020 for Abstracts checklist.	Abstract, p. 1, lines 1–21.
**INTRODUCTION**			
Rationale	3	Describe the rationale for the review in the context of existing knowledge.	Section 1, pp. 1–2, lines 24–46.
Objectives	4	Provide an explicit statement of the objective(s) or question(s) the review addresses.	Section 1, p. 2, lines 47–57 (review questions and organisation).
**METHODS**			
Eligibility criteria	5	Specify the inclusion and exclusion criteria for the review and how studies were grouped for the syntheses.	Section 2.2, p. 3, lines 83–103; the three linked evidence streams define the synthesis groups and transfer boundaries.
Information sources	6	Specify all databases, registers, websites, organisations, reference lists and other sources searched or consulted to identify studies. Specify the date when each source was last searched or consulted.	Section 2.1, pp. 2–3, lines 59–82 (Scopus, Web of Science Core Collection, search cut-off, and the reference-list/citation/publisher-record branch).
Search strategy	7	Present the full search strategies for all databases, registers and websites, including any filters and limits used.	Section 2.1 and Table 1, pp. 2–3, lines 59–76; database-specific wrappers, calendar-year bounds and query counts are in Supplementary Table S2, p. S11.
Selection process	8	Specify the methods used to decide whether a study met the inclusion criteria of the review, including how many reviewers screened each record and each report retrieved, whether they worked independently, and if applicable, details of automation tools used in the process.	Section 2.4, pp. 5–6, lines 194–207 (reviewer roles, sequential previous screen and incremental dual-database screen, full-text decision files and consensus). The automation aids used for record matching, deduplication and draft dispositions, and the fact that they were not substituted for the reviewers' decisions, are described at lines 199–206.
Data collection process	9	Specify the methods used to collect data from reports, including how many reviewers collected data from each report, whether they worked independently, any processes for obtaining or confirming data from study investigators, and if applicable, details of automation tools used in the process.	Section 2.4, p. 6, lines 208–222 (extracted variables, single-pass extraction without a duplicated per-item log, the recheck against the primary reports, and the automation aids used); Author Contributions, pp. 29–30, lines 1001–1006.
Data items	10a	List and define all outcomes for which data were sought. Specify whether all results that were compatible with each outcome domain in each study were sought (e.g. for all measures, time points, analyses), and if not, the methods used to decide which results to collect.	Review questions in Section 1, p. 2, lines 47–57; outcome domains and findings in Sections 3.2–3.3 and Tables 2–8, pp. 8–24.

PRISMA 2020 Checklist

Section and Topic	Item #	Checklist item	Location where item is reported
	10b	List and define all other variables for which data were sought (e.g. participant and intervention characteristics, funding sources). Describe any assumptions made about any missing or unclear information.	Extracted variables are specified in Section 2.4, p. 6, lines 208–222, and reported in Tables 2–7 and Supplementary Table S1, pp. S1–S11.
Study risk of bias assessment	11	Specify the methods used to assess risk of bias in the included studies, including details of the tool(s) used, how many reviewers assessed each study and whether they worked independently, and if applicable, details of automation tools used in the process.	Section 2.3, pp. 3–5, lines 104–193. Tailored M1–M5 and I1–I5 domains, rating definitions and their use in certainty downgrading are reported; completed ratings are in Supplementary Tables S3–S4, pp. S12–S16. The note preceding Table S3, p. S12, records that the appraisal was performed retrospectively by the two reviewers who extracted the studies and not by an assessor blinded to the synthesis.
Effect measures	12	Specify for each outcome the effect measure(s) (e.g. risk ratio, mean difference) used in the synthesis or presentation of results.	Not applicable—the review uses narrative synthesis of heterogeneous descriptive evidence and does not estimate intervention effects.
Synthesis methods	13a	Describe the processes used to decide which studies were eligible for each synthesis (e.g. tabulating the study intervention characteristics and comparing against the planned groups for each synthesis (item #5)).	Sections 2.2–2.4, pp. 3–6, lines 83–222; the evidence streams, E1–E4 tiers and applicable appraisal framework determine eligibility for each synthesis.
	13b	Describe any methods required to prepare the data for presentation or synthesis, such as handling of missing summary statistics, or data conversions.	Not applicable—no data conversions or imputation of missing summary statistics were undertaken.
	13c	Describe any methods used to tabulate or visually display results of individual studies and syntheses.	Results are displayed in Figures 1–3 and Tables 1–9, pp. 3–26, and Supplementary Tables S1–S4.
	13d	Describe any methods used to synthesize results and provide a rationale for the choice(s). If meta-analysis was performed, describe the model(s), method(s) to identify the presence and extent of statistical heterogeneity, and software package(s) used.	Narrative synthesis is organised by the two review questions (Section 1, p. 2, lines 47–57) and reported in Sections 3.2–3.3, pp. 6–23, with cross-study interpretation in Section 4, pp. 23–28.
	13e	Describe any methods used to explore possible causes of heterogeneity among study results (e.g. subgroup analysis, meta-regression).	No statistical heterogeneity analysis was applicable. Cross-study differences are explored in Sections 3.2.5 and 3.3.3, pp. 9–23, and Section 4, pp. 23–28.
	13f	Describe any sensitivity analyses conducted to assess robustness of the synthesized results.	No statistical meta-analysis was performed. The reference-list, citation and publisher-record sensitivity branch is described in Section 2.1, pp. 2–3, lines 77–82.
Reporting bias assessment	14	Describe any methods used to assess risk of bias due to missing results in a synthesis (arising from reporting biases).	No formal reporting-bias instrument was applicable. Possible publication, indexing and terminology biases are discussed under Review limitations, p. 28, lines 924–947.

PRISMA

PRISMA 2020 Checklist

Section and Topic	Item #	Checklist item	Location where item is reported
Certainty assessment	15	Describe any methods used to assess certainty (or confidence) in the body of evidence for an outcome.	Certainty methods and downgrade rules are defined in Section 2.3, pp. 3–5, lines 104–193; claim-level assessments are reported in Table 8, p. 24.
**RESULTS**			
Study selection	16a	Describe the results of the search and selection process, from the number of records identified in the search to the number of studies included in the review, ideally using a flow diagram.	Section 3.1, p. 6, lines 223–244, and the consolidated PRISMA flow diagram (Figure 1), p. 7.
	16b	Cite studies that might appear to meet the inclusion criteria, but which were excluded, and explain why they were excluded.	Figure 1, p. 7, and Section 3.1, p. 6, lines 226–244 (screening, retrieval and full-text exclusion counts/reasons). Avril et al. [34], excluded by publication type but retained as context, is identified on p. 3, lines 99–100.
Study characteristics	17	Cite each included study and present its characteristics.	Sections 3.2–3.3, pp. 6–23, cite and describe contributing studies in Tables 2–7; every included study and its role are itemised in Supplementary Table S1, pp. S1–S11.
Risk of bias in studies	18	Present assessments of risk of bias for each included study.	Study-level appraisals are in Supplementary Tables S3–S4, pp. S12–S16; Table 8, p. 24, reports their effects on claims. Table S1, pp. S1–S11, records the framework or coded non-applicability reason for every other included study.
Results of individual studies	19	For all outcomes, present, for each study: (a) summary statistics for each group (where appropriate) and (b) an effect estimate and its precision (e.g. confidence/credible interval), ideally using structured tables or plots.	Descriptive study findings are reported in Sections 3.2–3.3, pp. 6–23, and Tables 2–7; pooled effect estimates are not applicable.
Results of syntheses	20a	For each synthesis, briefly summarise the characteristics and risk of bias among contributing studies.	Study characteristics are reported in Sections 3.2–3.3 and Tables 2–7, pp. 6–23; evidence tier, appraisal limitation, consistency and certainty are summarised in Table 8, p. 24.
	20b	Present results of all statistical syntheses conducted. If meta-analysis was done, present for each the summary estimate and its precision (e.g. confidence/credible interval) and measures of statistical heterogeneity. If comparing groups, describe the direction of the effect.	No statistical synthesis or meta-analysis was conducted. Narrative synthesis is reported in Sections 3.2–3.3, pp. 6–23.
	20c	Present results of all investigations of possible causes of heterogeneity among study results.	Cross-study comparisons are reported in Sections 3.2.5 and 3.3.3, pp. 9–23, and interpreted in Section 4, pp. 23–28.
	20d	Present results of all sensitivity analyses conducted to assess the robustness of the synthesized results.	No statistical sensitivity analysis was conducted; the other-methods sensitivity branch is reported in Section 2.1, pp. 2–3, lines 77–82.
Reporting biases	21	Present assessments of risk of bias due to missing results (arising from reporting biases) for each synthesis assessed.	Potential publication, indexing and terminology biases are discussed under Review limitations, p. 28, lines 924–947.

PRISMA 2020 Checklist

Section and Topic	Item #	Checklist item	Location where item is reported
Certainty of evidence	22	Present assessments of certainty (or confidence) in the body of evidence for each outcome assessed.	Claim-level certainty assessments and their evidence bases are presented in Table 8, p. 24.
**DISCUSSION**			
Discussion	23a	Provide a general interpretation of the results in the context of other evidence.	Section 4, pp. 23–28, and Section 5, pp. 28–29.
	23b	Discuss any limitations of the evidence included in the review.	Table 8, p. 24, and Review limitations, p. 28, lines 924–947.
	23c	Discuss any limitations of the review processes used.	Sections 2.1–2.4, pp. 2–6, describe the review process; database, screening, appraisal and synthesis limitations are discussed under Review limitations, p. 28, lines 924–947.
	23d	Discuss implications of the results for practice, policy, and future research.	Section 4, pp. 23–28, and Section 5, pp. 28–29; established findings, recommendations and hypotheses are separated explicitly.
**OTHER INFORMATION**			
Registration and protocol	24a	Provide registration information for the review, including register name and registration number, or state that the review was not registered.	Section 2.1, p. 2, lines 59–62; OSF DOI 10.17605/OSF.IO/E3TGV.
	24b	Indicate where the review protocol can be accessed, or state that a protocol was not prepared.	Protocol access is reported in Section 2.1, p. 2, lines 59–62, and the Data Availability Statement, p. 30, lines 1020–1026; OSF DOI 10.17605/OSF.IO/E3TGV.
	24c	Describe and explain any amendments to information provided at registration or in the protocol.	The Open Science Framework registration records a protocol summary and does not specify information sources, databases or detailed eligibility criteria; these are reported in Sections 2.1–2.2 and Table 1, pp. 2–3, lines 59–103. No information provided at registration was amended. Web of Science query v was not executed separately because query vi is its strict superset (Table 1; Supplementary Table S2, p. S11).
Support	25	Describe sources of financial or non-financial support for the review, and the role of the funders or sponsors in the review.	Funding statement, p. 30, lines 1007–1010; funder-role statement in Conflicts of Interest, p. 30, lines 1035–1037.
Competing interests	26	Declare any competing interests of review authors.	Conflicts of Interest, p. 30, lines 1035–1037.
Availability of data, code and other materials	27	Report which of the following are publicly available and where they can be found: template data collection forms; data extracted from included studies; data used for all analyses; analytic code; any other materials used in the review.	Supplementary Materials and Data Availability, p. 30, lines 1011–1026. Tables S1–S4 accompany the article; the raw search and screening audit files are available from the corresponding author on reasonable request.

From: Page MJ, McKenzie JE, Bossuyt PM, Boutron I, Hoffmann TC, Mulrow CD, et al. The PRISMA 2020 statement: an updated guideline for reporting systematic reviews. BMJ 2021;372:n71. doi: 10.1136/bmj.n71. This work is licensed under CC BY 4.0. To view a copy of this license, visit https://creativecommons.org/licenses/by/4.0/
