# Supplementary material for: Functionally Graded Materials by Wire Arc Additive Manufacturing: Material-Pair Compatibility and Spatial Mechanical Characterisation—A Systematic Review
Source: Materials (Basel). 2026 Aug 8;19(16):3379. doi: 10.3390/ma19163379 (PMC13514652; doi:10.3390/ma19163379)
Supplement: Supplementary file 1 [file materials-19-03379-s001.zip › Supplementary.pdf]

Supplementary Materials

Functionally Graded Materials by Wire Arc Additive Manufacturing: Material-Pair Compatibility and Spatial Mechanical Characterisation—A Systematic Review

Manuscript ID: materials-4466069

Table S1. Study-level accounting for every included study

Every reference cited in the review is listed. **Included study** states whether the reference is counted among the included studies of the review. Studies carrying manufacturing or inverse-identification evidence into a synthesised claim are rated against M1–M5 or I1–I5 in Supplementary Tables S3 and S4; the **Appraisal** column gives the table in which each appears. For studies whose role is contextual, the claim-specific domains are not applicable and the **Code** column gives the reason, expanded below.

Codes for non-applicable appraisal domains

C1 Conventional or non-WAAM manufacturing route, cited to delimit the review scope.

C2 Characterisation-technique exemplar, cited to delimit the review scope.

C3 Foundational book, standard or general method reference, cited for context under Section 2.2 and not counted as an included study.

C4 Other contextual or scope-mapping source, including FGM classification, application sectors, adjacent AM/characterisation literature and methodological reviews not used as primary evidence in a synthesised claim.

Key	Reference	Year	Included study	Role	Framework	Appraisal	Code
Abdulmajeed2023	Abdulmajeed, A	2023	Y	Contextual	Not applicable	Not appraised	C4
Ahsan2020	Ahsan, M	2020	Y	Evidence-bearing	M1-M5	S3 (M1-M5)	—
Ahsan2021	Ahsan, M	2021	Y	Evidence-bearing	M1-M5	S3 (M1-M5)	—
Ahsan2024	Ahsan, M	2024	Y	Evidence-bearing	M1-M5	S3 (M1-M5)	—
AlHadrayi2022	Al-Hadrayi, Z	2022	Y	Contextual	Not applicable	Not appraised	C4
Amada1997	Amada, S	1997	N	Contextual	Not applicable	Not appraised	C4
AnilKumar2019	Anil Kumar, R	2019	Y	Contextual	Not applicable	Not appraised	C4
Ansari2021	Ansari, M	2021	N	Contextual	Not applicable	Not appraised	C4
Articek2013	Articek, U	2013	N	Contextual	Not applicable	Not appraised	C4
Ashby2011	Ashby, M	2011	N	Contextual	Not applicable	Not appraised	C3
Askarinejad2016	Askarinejad, S	2016	N	Contextual	Not applicable	Not appraised	C4
Astarita20211788	Astarita, A	2021	Y	Contextual	Not applicable	Not appraised	C4
ASTME82010	ASTM International	2025	N	Contextual	Not applicable	Not appraised	C3

Key	Reference	Year	Included study	Role	Framework	Appraisal	Code
Avril2008	Avril, S	2008	N	Contextual	Not applicable	Not appraised	C4
Ayan2022	Ayan, Y	2022	Y	Evidence-bearing	M1-M5	S3 (M1-M5)	—
Aysha2014	M	2014	N	Contextual	Not applicable	Not appraised	C4
Badoniya2024	Badoniya, P	2024	Y	Contextual	Not applicable	Not appraised	C4
Badoniya2025	Badoniya, P	2025	Y	Contextual	Not applicable	Not appraised	C4
Bai2018	Bai, R	2018	Y	Evidence-bearing	I1-I5	S4 (I1-I5)	—
Bastos2022	Bastos, R	2022	N	Contextual	Not applicable	Not appraised	C4
Bean2025	Bean, C	2025	Y	Evidence-bearing	I1-I5	S4 (I1-I5)	—
Bertin2016	Bertin, M	2016	Y	Evidence-bearing	I1-I5	S4 (I1-I5)	—
Besra2007	Besra, L	2007	Y	Contextual	Not applicable	Not appraised	C1
Bhavar2017	Bhavar, V	2017	N	Contextual	Not applicable	Not appraised	C4
Boettinger2000	Boettinger, W	2000	Y	Contextual	Not applicable	Not appraised	C1
Bourceret2025	Bourceret, A	2025	Y	Contextual	Not applicable	Not appraised	C4
Bruck2002	Bruck, H	2002	N	Contextual	Not applicable	Not appraised	C4
Bulla2023	Bulla, A	2023	Y	Evidence-bearing	M1-M5	S3 (M1-M5)	—
Campello2017	Campello, D	2017	Y	Contextual	Not applicable	Not appraised	C4
Chandrasekaran2020	Chandrasekaran, S	2020	Y	Evidence-bearing	M1-M5	S3 (M1-M5)	—
Chandrasekaran2022931	Chandrasekaran, S	2022	Y	Evidence-bearing	M1-M5	S3 (M1-M5)	—
Chen2020	Chen, B	2020	N	Contextual	Not applicable	Not appraised	C4
Chen2021	Chen, X	2021	Y	Evidence-bearing	M1-M5	S3 (M1-M5)	—
Chen2025	Chen, R	2025	Y	Contextual	Not applicable	Not appraised	C4
Chen2026	Chen, M	2026	Y	Evidence-bearing	I1-I5	S4 (I1-I5)	—
Chenglin1999	Chu, C	1999	Y	Contextual	Not applicable	Not appraised	C4
Choy2003	Choy, K	2003	Y	Contextual	Not applicable	Not appraised	C1
Chtourou2017228	Chtourou, R	2017	Y	Evidence-bearing	I1-I5	S4 (I1-I5)	—
Chung2006	Chung, Y	2006	N	Contextual	Not applicable	Not appraised	C3
Chung2006226	Chung, H	2006	N	Contextual	Not applicable	Not appraised	C4
Coget2024	Coget, Y	2024	N	Contextual	Not applicable	Not appraised	C4

Key	Reference	Year	Included study	Role	Framework	Appraisal	Code
Cognard2013	Cognard, J	2013	Y	Evidence-bearing	I1-I5	S4 (I1-I5)	—
Corallo2026	Corallo, L	2026	Y	Evidence-bearing	I1-I5	S4 (I1-I5)	—
Cunha2021	Cunha, F	2021	N	Contextual	Not applicable	Not appraised	C4
Cunha2026	Cunha, F	2026	Y	Evidence-bearing	M1-M5	S3 (M1-M5)	—
Domack2005	Domack, M	2005	N	Contextual	Not applicable	Not appraised	C4
Dong2017	Dong, B	2017	Y	Evidence-bearing	M1-M5	S3 (M1-M5)	—
Dorfman2005	Dorfman, M	2005	Y	Contextual	Not applicable	Not appraised	C1
Drenchev2002	Drenchev, L	2002	Y	Contextual	Not applicable	Not appraised	C1
Dutta2021	Dutta, S	2021	N	Contextual	Not applicable	Not appraised	C4
Dziri2025	Dziri, A	2025	Y	Evidence-bearing	M1-M5	S3 (M1-M5)	—
Ebhota2016	Ebhota, W	2016	Y	Contextual	Not applicable	Not appraised	C1
Farias2026	Farias, F	2026	Y	Contextual	Not applicable	Not appraised	C4
Ferreira2011	Ferreira, S	2011	Y	Contextual	Not applicable	Not appraised	C2
Fessler1997	Fessler, J	1997	Y	Contextual	Not applicable	Not appraised	C1
Fratini2005	Fratini, L	2005	N	Contextual	Not applicable	Not appraised	C4
Fu2024	Fu, J	2024	N	Contextual	Not applicable	Not appraised	C4
Fu2026	Fu, Z	2026	Y	Contextual	Not applicable	Not appraised	C4
Fukui1993156	Fukui, Y	1993	N	Contextual	Not applicable	Not appraised	C4
Ganesh2023	Ganesh, V	2023	Y	Contextual	Not applicable	Not appraised	C4
Gelin1995	Gelin, J	1995	Y	Contextual	Not applicable	Not appraised	C4
Geng2020	Geng, Y	2020	N	Contextual	Not applicable	Not appraised	C4
Geng20217406	Geng, Y	2021	Y	Contextual	Not applicable	Not appraised	C4
Ghaziani2021	Ghaziani, A	2021	N	Contextual	Not applicable	Not appraised	C4
Giljean2024	Giljean, S	2024	Y	Contextual	Not applicable	Not appraised	C4
Goncalves2024	Gonçalves, M	2024	Y	Contextual	Not applicable	Not appraised	C4
Gonzalez2025	Gonz\{}'alez, L	2025	Y	Contextual	Not applicable	Not appraised	C4

Key	Reference	Year	Included study	Role	Framework	Appraisal	Code
Guan2025	Guan, X	2025	Y	Contextual	Not applicable	Not appraised	C4
Gupta2015	Gupta, A	2015	N	Contextual	Not applicable	Not appraised	C4
Gupta2026	Gupta, A	2026	Y	Contextual	Not applicable	Not appraised	C4
Gushchina2023907	Gushchina, M	2023	Y	Contextual	Not applicable	Not appraised	C4
Hamill2026	Hamill, R	2026	Y	Evidence-bearing	I1-I5	S4 (I1-I5)	—
Han2022	Han, J	2022	Y	Evidence-bearing	M1-M5	S3 (M1-M5)	—
Han2025Mg	Han, Q	2025	Y	Evidence-bearing	M1-M5	S3 (M1-M5)	—
Hangai2012	Hangai, Y	2012	Y	Contextual	Not applicable	Not appraised	C2
Hangai2014	Hangai, Y	2014	Y	Contextual	Not applicable	Not appraised	C2
Hao201622	Hao, W	2016	N	Contextual	Not applicable	Not appraised	C4
Hao2016224	Hao, W	2016	N	Contextual	Not applicable	Not appraised	C4
Hazlehurst2014	Hazlehurst, K	2014	N	Contextual	Not applicable	Not appraised	C4
He2021	He, Z	2021	N	Contextual	Not applicable	Not appraised	C4
Henriques2023Arcan	Henriques, J	2023	N	Contextual	Not applicable	Not appraised	C4
Hoan2026	Hoan, T	2026	Y	Contextual	Not applicable	Not appraised	C4
Hou2025	Hou, T	2025	Y	Evidence-bearing	I1-I5	S4 (I1-I5)	—
Hrairi2011	Hrairi, M	2011	N	Contextual	Not applicable	Not appraised	C4
Hu2006	Hu, Y	2006	N	Contextual	Not applicable	Not appraised	C4
Huang2007	Huang, M	2007	N	Contextual	Not applicable	Not appraised	C4
Huang2026	Huang, Y	2026	Y	Contextual	Not applicable	Not appraised	C4
ISO17296-22015	International Organization for Standardization	2015	N	Contextual	Not applicable	Not appraised	C3
Jain2014	Jain, A	2014	N	Contextual	Not applicable	Not appraised	C4
jbi2024manual	JBI	2024	N	Contextual	Not applicable	Not appraised	C4
Jedamzik2000	Jedamzik, R	2000	Y	Contextual	Not applicable	Not appraised	C1
Jeong2024	Jeong, Y	2024	Y	Contextual	Not applicable	Not appraised	C4
Jia2017	Jia, Q	2017	Y	Evidence-bearing	I1-I5	S4 (I1-I5)	—
Jiang2020	Jiang, H	2020	Y	Evidence-bearing	I1-I5	S4 (I1-I5)	—

Key	Reference	Year	Included study	Role	Framework	Appraisal	Code
John2026	John, D	2026	Y	Evidence-bearing	M1-M5	S3 (M1-M5)	—
Jones2024DIC	Jones, E	2024	Y	Evidence-bearing	I1-I5	S4 (I1-I5)	—
Jullien2025	Jullien, M	2025	Y	Evidence-bearing	M1-M5	S3 (M1-M5)	—
K.Harsha2006	K	2006	Y	Contextual	Not applicable	Not appraised	C1
Kabaldin2023	Kabaldin, Y	2023	Y	Contextual	Not applicable	Not appraised	C4
Katayama2011	Katayama, T	2011	Y	Contextual	Not applicable	Not appraised	C1
Khalfallah2014	Khalfallah, A	2014	Y	Evidence-bearing	I1-I5	S4 (I1-I5)	—
Khan2019	Khan, T	2019	N	Contextual	Not applicable	Not appraised	C4
Kim2013	Kim, J	2013	Y	Contextual	Not applicable	Not appraised	C4
Kim2019	Kim, D	2019	N	Contextual	Not applicable	Not appraised	C4
Kim2023245	Kim, C	2023	Y	Evidence-bearing	I1-I5	S4 (I1-I5)	—
Koko2026	Koko, A	2026	Y	Contextual	Not applicable	Not appraised	C4
Kosco2026	Kosco, T	2026	Y	Evidence-bearing	I1-I5	S4 (I1-I5)	—
Kreuz2001	Kreuz, P	2001	N	Contextual	Not applicable	Not appraised	C4
Kumar2010	Kumar, S	2010	N	Contextual	Not applicable	Not appraised	C4
Lai2024	Lai, T	2024	Y	Contextual	Not applicable	Not appraised	C4
Larichkin2025	Larichkin, A	2025	Y	Evidence-bearing	I1-I5	S4 (I1-I5)	—
Lattanzi2020	Lattanzi, A	2020	Y	Evidence-bearing	I1-I5	S4 (I1-I5)	—
Launey20092919	Launey, M	2009	N	Contextual	Not applicable	Not appraised	C4
Leu2012	Leu, M	2012	N	Contextual	Not applicable	Not appraised	C4
Lezgy-Nazargah2015154	Lezgy-Nazargah, M	2015	N	Contextual	Not applicable	Not appraised	C4
Li2002	Li, L	2002	N	Contextual	Not applicable	Not appraised	C4
Li2022	Li, T	2022	Y	Evidence-bearing	M1-M5	S3 (M1-M5)	—
Li2022461	Li, T	2022	Y	Evidence-bearing	M1-M5	S3 (M1-M5)	—
Li2023	Li, T	2023	Y	Evidence-bearing	M1-M5	S3 (M1-M5)	—
Li202343	Li, T	2023	Y	Evidence-bearing	M1-M5	S3 (M1-M5)	—
Liang2023	Liang, L	2023	N	Contextual	Not applicable	Not appraised	C4
Liu2017	Liu, Z	2017	N	Contextual	Not applicable	Not appraised	C4
Liu2026	Liu, W	2026	Y	Contextual	Not applicable	Not appraised	C4
Liu2026a	Liu, F	2026	Y	Contextual	Not applicable	Not appraised	C4

Key	Reference	Year	Included study	Role	Framework	Appraisal	Code
Liu2026HEA	Liu, C	2026	Y	Evidence-bearing	M1-M5	S3 (M1-M5)	—
Lu2021	Lu, Y	2021	N	Contextual	Not applicable	Not appraised	C4
Lu2021TA1	Lu, L	2021	Y	Evidence-bearing	M1-M5	S3 (M1-M5)	—
Lucignano2009	Lucignano, C	2009	Y	Contextual	Not applicable	Not appraised	C4
MA2015august	Ma, Y	2015	Y	Evidence-bearing	M1-M5	S3 (M1-M5)	—
MA2015february	Ma, Y	2015	Y	Evidence-bearing	M1-M5	S3 (M1-M5)	—
Madhu2025	Madhu, M	2025	Y	Contextual	Not applicable	Not appraised	C4
Mahamood2012	Mahamood, R	2012	N	Contextual	Not applicable	Not appraised	C4
Mahamood2017	Mahamood, R	2017	N	Contextual	Not applicable	Not appraised	C3
Malik2026	Malik, R	2026	Y	Evidence-bearing	I1-I5	S4 (I1-I5)	—
Marinelli2019	Marinelli, G	2019	Y	Evidence-bearing	M1-M5	S3 (M1-M5)	—
Markiewicz2017	Markiewicz, E	2017	Y	Contextual	Not applicable	Not appraised	C4
Marola2026	Marola, S	2026	Y	Contextual	Not applicable	Not appraised	C4
Martins2018330	Martins, J	2018	N	Contextual	Not applicable	Not appraised	C4
Marwan2023	El-Husseiny, M	2023	Y	Evidence-bearing	M1-M5	S3 (M1-M5)	—
Maskery2016	Maskery, I	2016	N	Contextual	Not applicable	Not appraised	C4
Mei2026	Mei, M	2026	Y	Contextual	Not applicable	Not appraised	C4
MiKaRaKaFo99	Miyamoto, Y	1999	N	Contextual	Not applicable	Not appraised	C3
Milosevic2016	Milosevi\{}'c, M	2016	Y	Evidence-bearing	I1-I5	S4 (I1-I5)	—
Mishra2016	Mishra, A	2016	N	Contextual	Not applicable	Not appraised	C4
Monnet2026	Monnet, A	2026	Y	Evidence-bearing	M1-M5	S3 (M1-M5)	—
Naebe2016	Naebe, M	2016	N	Contextual	Not applicable	Not appraised	C4
Najem2026	Najem, A	2026	Y	Contextual	Not applicable	Not appraised	C4
Nakata2012	Nakata, T	2012	Y	Evidence-bearing	I1-I5	S4 (I1-I5)	—
Nayakanti2013	Nayakanti, N	2013	Y	Evidence-bearing	I1-I5	S4 (I1-I5)	—
Nazemi2019	Nazemi, N	2019	Y	Evidence-bearing	I1-I5	S4 (I1-I5)	—
Nemat-Alla2011	Nemat-Alla, M	2011	Y	Contextual	Not applicable	Not appraised	C1
Ni2026	Ni, Y	2026	N	Contextual	Not applicable	Not appraised	C4
Nigamaa2014295	Nigamaa, N	2014	N	Contextual	Not applicable	Not appraised	C4
Niino1987	Niino, M	1987	N	Contextual	Not applicable	Not appraised	C4

Key	Reference	Year	Included study	Role	Framework	Appraisal	Code
Nimer2019	Nimer, S	2019	Y	Evidence-bearing	I1-I5	S4 (I1-I5)	—
Pacheco2020	Pacheco, M	2020	N	Contextual	Not applicable	Not appraised	C4
Pelz2024	Pelz, J	2024	Y	Contextual	Not applicable	Not appraised	C4
Pierron2020e12370	Pierron, F	2021	N	Contextual	Not applicable	Not appraised	C4
Pierron202359	Pierron, F	2023	N	Contextual	Not applicable	Not appraised	C4
Prates2016547	Prates, P	2016	Y	Contextual	Not applicable	Not appraised	C4
PRISMA2021	Page, M	2020	N	Contextual	Not applicable	Not appraised	C4
Qi2019	Qi, S	2019	N	Contextual	Not applicable	Not appraised	C4
Qiao2013	Qiao, Z	2013	Y	Contextual	Not applicable	Not appraised	C1
Que2023	Que, Y	2023	Y	Evidence-bearing	I1-I5	S4 (I1-I5)	—
Rajan2008	Rajan, T	2008	Y	Contextual	Not applicable	Not appraised	C1
RajeshKannan2020	Rajesh~Kannan, A	2020	Y	Evidence-bearing	M1-M5	S3 (M1-M5)	—
Reisgen2019	Reisgen, U	2019	Y	Evidence-bearing	M1-M5	S3 (M1-M5)	—
Rodrigues2019	Rodrigues, T	2019	N	Contextual	Not applicable	Not appraised	C4
Rodrigues2021	Rodrigues, T	2022	Y	Evidence-bearing	M1-M5	S3 (M1-M5)	—
Rodrigues2022237	Rodrigues, T	2022	Y	Evidence-bearing	M1-M5	S3 (M1-M5)	—
Rossi2012	Rossi, M	2012	Y	Evidence-bearing	I1-I5	S4 (I1-I5)	—
Sakaguchi2026Creep	Sakaguchi, M	2026	Y	Evidence-bearing	I1-I5	S4 (I1-I5)	—
Sakaridis2024	Sakaridis, E	2024	Y	Contextual	Not applicable	Not appraised	C4
Saranath2015	Saranath, K	2015	Y	Evidence-bearing	I1-I5	S4 (I1-I5)	—
Sasikumar2022230	Sasikumar, R	2022	Y	Evidence-bearing	M1-M5	S3 (M1-M5)	—
Sburlati20133649	Sburlati, R	2013	N	Contextual	Not applicable	Not appraised	C4
Schneider-Maunoury2019171	Schneider-Maunoury, C	2019	Y	Contextual	Not applicable	Not appraised	C4
Schroeder2026	Schroeder, N	2026	Y	Evidence-bearing	I1-I5	S4 (I1-I5)	—
Schwartz2004	Schwartz, R	2004	Y	Contextual	Not applicable	Not appraised	C1
Scott2012	Scott, J	2012	N	Contextual	Not applicable	Not appraised	C4
Semenchuk20241969	Semenchuk, V	2024	Y	Contextual	Not applicable	Not appraised	C4
Senthil2021661	Senthil, T	2021	Y	Evidence-bearing	M1-M5	S3 (M1-M5)	—
Senthil2022	Senthil, T	2022	Y	Evidence-bearing	M1-M5	S3 (M1-M5)	—
SerranoMunoz2025	Serrano-Munoz, I	2025	Y	Contextual	Not applicable	Not appraised	C4

Key	Reference	Year	Included study	Role	Framework	Appraisal	Code
Shahmirzaloo2021	Shahmirzaloo, A	2021	Y	Evidence-bearing	I1-I5	S4 (I1-I5)	—
Shalnova2022	Shalnova, S	2022	Y	Contextual	Not applicable	Not appraised	C4
Shen2015	Shen, C	2015	Y	Evidence-bearing	M1-M5	S3 (M1-M5)	—
Shen2016	Shen, C	2016	Y	Evidence-bearing	M1-M5	S3 (M1-M5)	—
Shen2018411	Shen, C	2018	Y	Contextual	Not applicable	Not appraised	C4
Shen2020january	Shen, C	2020	Y	Evidence-bearing	M1-M5	S3 (M1-M5)	—
Shen2020july	Shen, C	2020	Y	Evidence-bearing	M1-M5	S3 (M1-M5)	—
Shen2021	Shen, C	2021	Y	Evidence-bearing	M1-M5	S3 (M1-M5)	—
Shin2025	Shin, D	2025	Y	Contextual	Not applicable	Not appraised	C4
Sidorov2024	Sidorov, I	2024	Y	Contextual	Not applicable	Not appraised	C4
Silva2021	Silva, M	2021	Y	Contextual	Not applicable	Not appraised	C4
Singh2021	Dev Singh, D	2021	N	Contextual	Not applicable	Not appraised	C4
Singh2024	Singh, M	2024	Y	Evidence-bearing	M1-M5	S3 (M1-M5)	—
Smith2025674	Smith, D	2025	Y	Contextual	Not applicable	Not appraised	C4
Song2007	Song, Y	2007	Y	Contextual	Not applicable	Not appraised	C1
Srinivasan2016	Srinivasan, N	2016	Y	Evidence-bearing	I1-I5	S4 (I1-I5)	—
Srivastava2015	Srivastava, M	2015	N	Contextual	Not applicable	Not appraised	C4
Stanisz2022	Stanisz, D	2022	Y	Evidence-bearing	I1-I5	S4 (I1-I5)	—
Starman2025	Starman, B	2025	Y	Contextual	Not applicable	Not appraised	C4
Stewart2026	Stewart, C	2026	Y	Evidence-bearing	I1-I5	S4 (I1-I5)	—
Stutz2011	Stutz, S	2011	Y	Evidence-bearing	I1-I5	S4 (I1-I5)	—
Sudarmadji2011	Sudarmadji, N	2011	N	Contextual	Not applicable	Not appraised	C4
Sun20154358	Sun, G	2015	Y	Evidence-bearing	I1-I5	S4 (I1-I5)	—
Sun2025	Sun, J	2025	Y	Contextual	Not applicable	Not appraised	C4
Suresh1997	Suresh, S	1997	N	Contextual	Not applicable	Not appraised	C4
Sutton2008	Sutton, M	2008	Y	Evidence-bearing	I1-I5	S4 (I1-I5)	—
Tang2026	Tang, R	2026	Y	Contextual	Not applicable	Not appraised	C4
Teja2020808	Teja, K	2020	Y	Contextual	Not applicable	Not appraised	C4
Thapliyal2026	Thapliyal, S	2026	Y	Contextual	Not applicable	Not appraised	C4
Tian2023	Tian, H	2023	N	Contextual	Not applicable	Not appraised	C4

Key	Reference	Year	Included study	Role	Framework	Appraisal	Code
Tomar2026	Tomar, B	2026	Y	Evidence-bearing	I1-I5	S4 (I1-I5)	—
Traini2008	Traini, T	2008	N	Contextual	Not applicable	Not appraised	C4
Val2017	Val, J	2017	N	Contextual	Not applicable	Not appraised	C4
Valmalle2026	Valmalle, M	2026	Y	Evidence-bearing	I1-I5	S4 (I1-I5)	—
Varma1998	Varma, A	1998	Y	Contextual	Not applicable	Not appraised	C1
VFMPierron	Pierron, F	2012	N	Contextual	Not applicable	Not appraised	C3
Vijay2019	Vijay, S	2019	Y	Contextual	Not applicable	Not appraised	C2
Wang2018	Wang, J	2018	Y	Evidence-bearing	M1-M5	S3 (M1-M5)	—
Wang20217472	Wang, J	2021	Y	Contextual	Not applicable	Not appraised	C4
Wang2025ML	Wang, X	2025	Y	Evidence-bearing	M1-M5	S3 (M1-M5)	—
Wang2026HAZ	Wang, Y	2026	Y	Evidence-bearing	I1-I5	S4 (I1-I5)	—
Watanabe2001	Watanabe, Y	2001	Y	Contextual	Not applicable	Not appraised	C2
Watanabe2009	Watanabe, Y	2009	Y	Contextual	Not applicable	Not appraised	C1
Wilson2012	Wilson, J	2012	Y	Contextual	Not applicable	Not appraised	C1
Withers2021	Withers, P	2021	Y	Contextual	Not applicable	Not appraised	C2
Xavier2007	Xavier, J	2007	N	Contextual	Not applicable	Not appraised	C4
Xavier2018556	Xavier, J	2018	N	Contextual	Not applicable	Not appraised	C4
Xu2026	Xu, Y	2026	Y	Contextual	Not applicable	Not appraised	C4
Yadav2025	Yadav, A	2025	Y	Evidence-bearing	I1-I5	S4 (I1-I5)	—
Yan2023HAZ	Yan, R	2023	Y	Evidence-bearing	I1-I5	S4 (I1-I5)	—
Yeo1998	Yeo, J	1998	Y	Contextual	Not applicable	Not appraised	C1
Yoo2023	Yoo, S	2023	Y	Evidence-bearing	M1-M5	S3 (M1-M5)	—
Yoon2017	Yoon, S	2017	N	Contextual	Not applicable	Not appraised	C4
Yu2021	Yu, L	2022	Y	Evidence-bearing	M1-M5	S3 (M1-M5)	—
Yu2023	Yu, X	2023	Y	Evidence-bearing	M1-M5	S3 (M1-M5)	—
Yu2026	Yu, B	2026	Y	Contextual	Not applicable	Not appraised	C4
Yuan2018	Yuan, Y	2018	N	Contextual	Not applicable	Not appraised	C4
Yue2015	Yue, Z	2015	Y	Evidence-bearing	I1-I5	S4 (I1-I5)	—
Zeng2020	Zeng, Z	2020	Y	Evidence-bearing	M1-M5	S3 (M1-M5)	—
Zhang20102304	Zhang, C	2010	N	Contextual	Not applicable	Not appraised	C4

Key	Reference	Year	Included study	Role	Framework	Appraisal	Code
Zhang2018	Zhang, J	2018	Y	Contextual	Not applicable	Not appraised	C2
Zhang2019	Zhang, C	2019	N	Contextual	Not applicable	Not appraised	C4
Zhang2019PostNecking	Zhang, H	2019	Y	Evidence-bearing	I1-I5	S4 (I1-I5)	—
Zhang2022	Zhang, Y	2022	N	Contextual	Not applicable	Not appraised	C4
Zhang20221007	Zhang, J	2022	Y	Evidence-bearing	M1-M5	S3 (M1-M5)	—
Zhang20221016	Zhang, J	2022	Y	Evidence-bearing	M1-M5	S3 (M1-M5)	—
Zhang20229703	Zhang, J	2022	Y	Evidence-bearing	M1-M5	S3 (M1-M5)	—
Zhang2023	Zhang, J	2023	Y	Evidence-bearing	M1-M5	S3 (M1-M5)	—
Zhao2019	Zhao, W	2019	N	Contextual	Not applicable	Not appraised	C4
Zhou2022Explosive	Zhou, Q	2022	Y	Evidence-bearing	M1-M5	S3 (M1-M5)	—
Zhou2025Pipe	Zhou, H	2025	Y	Evidence-bearing	M1-M5	S3 (M1-M5)	—
Zhu2026	Zhu, Y	2026	Y	Evidence-bearing	I1-I5	S4 (I1-I5)	—

Table S2. Query-by-query database result counts

Scopus and Web of Science Core Collection were searched for eligible publications up to and including 31 July 2026. The query bodies are reproduced in Table 1 of the main manuscript. The database interfaces implemented calendar-year bounds: Scopus applied `TITLE-ABS-KEY(...) AND PUBYEAR < 2027`, whereas Web of Science applied `TS=(...) AND PY=(1900-2026)`. These year-level bounds do not replace the stated day-specific eligibility cut-off. Counts are raw occurrences from the separately exported, intentionally overlapping queries and are not numbers of unique records. Web of Science query v was not separately executed because query vi contains its complete term set plus one additional `OR` term.

Query	Scopus, n	WoS, n	Execution note
i	32	2	Executed in both databases
ii	10	8	Executed in both databases
iii	11	12	Executed in both databases
iv	11	1	Executed in both databases
v	3	—	Not separately executed in Web of Science; subsumed by query vi
vi	54	26	Executed in both databases
vii	98	26	Executed in both databases
viii	106	87	Executed in both databases
ix	589	573	Executed in both databases
**Total raw occurrences**	**914**	**735**	Scopus total from nine exports; Web of Science total from eight exports

Supplementary Tables S3–S4. Study-level critical appraisal

Tables S3 and S4 report the application of the appraisal domains defined in Section 2.3 of the main manuscript to every included study that contributes evidence to a synthesised claim. Manufacturing evidence is rated against M1–M5 and inverse-identification or full-field evidence against I1–I5. Each study also carries its evidence tier (E1–E4). The 51 manufacturing rows and 43 inverse/full-field rows comprise 94 evidence-bearing studies; the remaining 82 of the 176 included studies serve scope-mapping or contextual roles for which these claim-specific domains are not applicable and are identified in Table S1.

Four points govern the reading of these tables. First, the ratings are recorded per study and are not summed; no ranking of studies is implied. Secondly, "unclear" denotes that the domain could not be resolved from the fields extracted for this review; it is not a finding that the primary study failed to report the item. Thirdly, "not met" is used only where the domain applies to the study design and the report is silent on it. Fourthly, the appraisal was performed retrospectively by the two reviewers who extracted the studies and not by an assessor blinded to the synthesis. Bracketed monospaced identifiers are the bibliography keys used in the main manuscript and in Table S1.

Table S3. Study-level appraisal of the manufacturing evidence against domains M1–M5. F, met; P, partly met; N, not met; U, unclear (not resolvable from the extracted fields). Tiers: E1, deliberate compositional gradient by a wire-fed arc process; E2, other metal AM, bimetallic or sequentially deposited arc build, or arc process requiring a non-wire feedstock; E3, indirect experimental gradient or dissimilar-joint evidence.

Study	System	Tier	M1	M2	M3	M4	M5	Governing appraisal note
Shen et al. [`Shen2015`]	Fe–Al	E1	F	F	F	P	P	Composition path set and reported over 15–55 at.% Al; phases identified; mechanical reporting without replicate or scatter information
Shen et al. [`Shen2016`]	Fe_3Al	E2	F	F	F	F	F	Fixed composition verified as uniform; longitudinal and transverse tensile response reported and attributed to epitaxial growth
Shen et al. [`Shen2020january`]	Fe–Ni	E1	F	F	F	U	P	Phase and lattice evolution resolved by microscopy and neutron diffraction; mechanical reporting not resolvable from the extracted fields
Shen et al. [`Shen2020july`]	Fe–Ni	E1	F	F	F	U	P	As above; the study addresses phase evolution rather than mechanical response
Shen et al. [`Shen2021`]	Fe–Ni	E1	F	F	F	U	P	Composition path 20–50% Ni verified; no spatial mechanical law reported
Wang et al. [`Wang2018`]	Ti–Al	E1	F	F	P	U	P	Dual-wire feed control of the path documented; phase identification limited in the extracted fields
Ma et al. [`MA2015august`]	γ-TiAl	E2	F	P	F	U	F	Fixed nominal composition; interpass temperature linked to thermal history and microstructure
Ma et al. [`MA2015february`]	γ-TiAl	E2	F	P	F	U	F	Phase identification reported; mechanical reporting not resolvable
Zeng et al. [`Zeng2020`]	Ni–Ti	E1	F	P	F	F	F	Austenitic phase confirmed and tensile superelasticity reported; composition verification along the path limited
Yu et al. [`Yu2021`]	NiTi wall	E2	F	P	F	F	F	Layerwise thermal history related to phase transformation and mechanical response at fixed nominal composition
Dong et al. [`Dong2017`]	Cu–Al	E1	F	P	F	U	P	Feedstock and shielding fully specified; $CuAl_2$ and Cu_9Al_4 identified; realised composition path not verified in the extracted fields
Cunha et al. [`Cunha2026`]	Cu–CuAl library	E1	F	F	P	F	F	Composition library with hardness, eddy-current and tensile testing supported by DIC; strength–ductility trade-off mapped across the gradient
Marinelli et al. [`Marinelli2019`]	Ta–Mo–W	E1	F	F	F	P	P	Composition and hardness profiles reported; mechanical evidence limited to hardness, and thermal expansion is not isolated from phase and process effects
Rodrigues et al. [`Rodrigues2021`]	HSLA–Cu-Al	E1	F	P	F	F	F	Synchrotron XRD with hardness, conductivity and tensile data along the build height
Zhang et al. [`Zhang2023`]	IN625–HSLA	E1	F	P	P	F	F	In-situ heat treatment; build-direction strength reported with scatter
Zhang et al. [`Zhang20229703`]	IN625–HSLA	E1	F	P	P	U	P	Reported collectively with the companion studies; individual mechanical attribution not resolvable
Zhang et al. [`Zhang20221016`]	IN625–HSLA	E1	F	P	F	F	F	Electromagnetic stirring related to dendrite spacing and tensile strength

Study	System	Tier	M1	M2	M3	M4	M5	Governing appraisal note
Zhang et al. [Zhang20221007]	IN625–HSLA	E1	F	F	P	P	F	Optimised transition strategy defined against an identified weak composition interval
Han et al. [Han2022]	Ti6Al4V–IN625	E1	F	F	U	P	P	Full composition path reported; compression data for vertical samples only; phase identification not resolvable
Chen et al. [Chen2021]	Ti6Al4V–SS316L	E1	F	F	F	P	F	Fe_2Ti identified in the cracked interval and hardness maximum located; mechanical evidence limited to hardness
Ahsan et al. [Ahsan2021]	IN625–SS316L	E2	F	P	F	F	F	Bimetallic build; interfacial growth and diffusion characterised with hardness, tensile and fractographic data
Sasikumar et al. [Sasikumar2022230]	IN625–SS316L	E2	F	U	U	U	U	Included in the material-system map; domains not resolvable from the extracted fields
Rodrigues et al. [Rodrigues2022237]	IN625–SS316L	E2	F	U	U	U	U	As above
Yu et al. [Yu2023]	SS316L–IN625	E1	F	F	F	F	F	Two composition increments compared; austenitic phase, absence of cracking, microhardness, tensile response and fracture position all reported
Li et al. [Li2022]	SS308L–IN625	E1	F	F	F	F	F	Composition, microstructure, phases and mechanical properties reported across the transition
Li et al. [Li2022461]	SS308L–IN625	E1	F	P	F	P	F	Crack type, location, initiation and propagation characterised; mechanical reporting secondary to the defect analysis
Li et al. [Li2023]	SS308L–IN625	E1	F	F	P	F	F	Weak 10–50% interval identified and strengthened by local Nb alloying, with strength and elongation reported before and after
Li et al. [Li202343]	SS308L–IN625	E1	F	P	F	F	F	Two wire-feeding strategies compared for geometry, microstructure and mechanical properties
Yoo et al. [Yoo2023]	SS304L–IN625	E1	F	P	P	F	F	WAAM and hybrid LMD–WAAM routes compared; fracture relocation to an internal composition interface reported
Chandrasekaran et al. [Chandrasekaran2020]	DSS–CMS	E2	F	P	F	F	F	X-ray CT, tensile, microstructure, EDX, microhardness, fractography and corrosion testing on a sequential build
Chandrasekaran et al. [Chandrasekaran2022931]	DSS–CMS	E2	F	U	U	U	U	Companion report; domains not resolvable from the extracted fields
Ahsan et al. [Ahsan2020]	LCS–SS316L	E2	F	P	F	P	F	Heat treatment shifted the failure site; SEM, EDX and hardness reported, tensile reporting limited
Ayan and Kahraman [Ayan2022]	LAS–ASS	E2	F	P	F	F	F	Interleaved architecture rather than a monotonic path; XRD, hardness, tensile and fatigue data reported
Rajesh Kannan et al. [RajeshKannan2020]	SS904L–Hastelloy C-276	E2	F	P	P	F	F	Sequential build; yield, tensile and fatigue strengths reported with the failure region identified
Senthil et al. [Senthil2022]	IN825–SS316L	E2	F	U	U	U	U	Included in the material-system map; domains not resolvable from the extracted fields
Senthil et al. [Senthil2021661]	IN825–SS316L	E2	F	U	U	U	U	As above
Lu et al. [Lu2021TA1]	TA1–IN625	E1	F	P	P	P	F	Vickers hardness correlated with microstructure across the transition; mechanical evidence limited to hardness
Marwan et al. [Marwan2023]	HSLA–Si-Bronze	E2	F	P	F	F	F	Diffusion zone characterised with hardness, tensile, bending and shear data
Singh et al. [Singh2024]	Ni–Cr–Mo	E1	F	P	F	F	F	Hardness rise attributed to identified precipitates; tensile trend reported in the same direction
John et al. [John2026]	SS316L–IN718	E1	F	F	F	N	F	Composition path confirmed by optical emission spectroscopy and a single austenitic matrix by XRD; tensile properties explicitly not reported
Wang et al. [Wang2025ML]	P91–IN740H	E1	F	F	F	P	F	High-throughput hardness, porosity, composition and microscopy data set; the authors report scale-transfer uncertainty in tensile response

Study	System	Tier	M1	M2	M3	M4	M5	Governing appraisal note
Han et al. [Han2025Mg]	Mg-Al-Si–Mg-Gd-Y-Zn	E2	F	F	F	F	F	Transition width and elemental profile measured; hardness, strength, elongation and fracture location all reported
Reisgen et al. [Reisgen2019]	Multi-wire steels	E1	F	P	U	U	P	Demonstrates melt-pool composition control by wire-feed speed; microstructural and mechanical reporting not resolvable
Liu et al. [Liu2026HEA]	HEA–ER120S-G	E2	F	F	F	U	F	Wire–powder hybrid process; phase constitution, grain orientation and corrosion response controlled spatially; mechanical reporting not resolvable
Monnet et al. [Monnet2026]	Fe–Cr–C hardfacing/S355MC	E3	F	F	F	F	F	Through-thickness microstructure linked to quasi-static and dynamic constitutive response; hardfacing gradient rather than WAAM composition control
Zhou et al. [Zhou2025Pipe]	TA10/6061 explosive-weld pipe	E3	F	P	P	F	F	Interface and axial-compression performance characterised; no additive manufacture or programmed path
Dziri et al. [Dziri2025]	Oxygen-graded titanium	E3	F	F	F	F	F	Measured oxygen gradient related to local elastic properties; gradient produced by oxygen uptake rather than wire-feed control
Jullien et al. [Jullien2025]	Oxidised Alloy 718	E3	F	F	F	F	F	Local testing resolves an oxidation-induced property gradient; indirect for designed metal–metal gradients
Ahsan et al. [Ahsan2024]	SS316L–IN625 heterogeneous DED	E2	F	F	F	F	F	Compositional and microstructural heterogeneity linked to strength–ductility response; not a continuous identified property law
Bulla et al. [Bulla2023]	Equiatomic NiTi twin-wire DED-arc	E2	F	F	F	F	F	Local thermal-cycle effects resolved at fixed target composition; not a programmed gradient
Zhou et al. [Zhou2022Explosive]	TA2/Q235 explosive-weld interface	E3	F	F	F	F	F	Interface microstructure and dynamic-shear response reported; indirect dissimilar-joint evidence

Table S4. Study-level appraisal of the inverse-identification and full-field evidence against domains I1–I5. Ratings as in Supplementary Table S3. Tiers: E1, deliberate compositional gradient; E2, other metal AM; E3, indirect experimental evidence from a weld, local-gradient material or heterogeneous test; E4, numerical or synthetic data without a physical identification experiment. No study combines tier E1 with a met rating on I3–I5.

Study	Specimen	Tier	I1	I2	I3	I4	I5	Governing appraisal note
Malik et al. [Malik2026]	T-WAAM IN625–SS308L	E1	F	P	N	N	N	DIC on a deliberate interlayer; no constitutive model defined and no identification attempted
Tomar et al. [Tomar2026]	CMT-WAAM SS316L–Cu	E1	F	P	N	N	N	DIC locates fracture initiation; no parameter field identified
Bean et al. [Bean2025]	Composition-gradient coupon	E1	F	F	P	P	P	High-resolution DIC with data-driven mapping to a fatigue indicator; a derived local indicator rather than an identified constitutive law
Stewart et al. [Stewart2026]	AM 316L–IN718 interface	E1	F	P	N	N	N	Notch placement and DIC; strength and toughness reported without identification
Chen et al. [Chen2026]	DED M247/IN718	E2	F	P	N	N	N	DIC confirms coordinated deformation between hard and soft domains; no inversion performed
Corallo et al. [Corallo2026]	LPBF 316L	E2	F	P	F	P	P	FEMU-assisted calibration motivating a Cazacu–Barlat model; homogeneous specimen
Valmalle et al. [Valmalle2026]	LPBF AlSi10Mg lattice	E2	F	F	F	P	F	Displacement uncertainty quantified at ≈0.05 voxel; recovered strut properties compared against ex-situ dog-bone values, revealing a scale effect
Nazemi and Masoumi Khalilabad [Nazemi2019]	Aluminium welded joint	E3	P	U	F	U	U	FEMU for HAZ strength variation; measurement uncertainty and identifiability not resolvable
Kim et al. [Kim2023245]	FSW AA6061-T6	E3	F	P	F	P	P	VFM with DIC and FEM for local yield stress on an incidental gradient
Sutton et al. [Sutton2008]	FSW 2024-T3	E3	P	P	F	P	U	Elastic and plastic properties identified; independent validation not resolvable
Saranath and Ramji [Saranath2015]	Ti–6Al–4V EBW	E3	F	P	F	P	U	VFM with DIC across a weld gradient
Shahmirzaloo et al. [Shahmirzaloo2021]	FSW 2024-T3	E3	F	P	F	P	U	Local properties identified; uncertainty reporting limited
Jiang et al. [Jiang2020]	Al-Mg laser weld	E3	F	P	F	P	U	Local elasto-plastic properties from DIC fields
Bai et al. [Bai2018]	Aluminium laser weld	E3	F	P	F	P	P	Kinematic hardening parameters identified with FEM support
Koščo and Chmelko [Kosco2026]	S355 weld region	E3	F	P	F	P	U	DIC strains along the notched minimum section with a force-residual objective
Hou et al. [Hou2025]	Superalloy weld micro-zones	E3	F	P	F	P	F	Indentation and FE inversion validated against DIC from 20 to 800 °C; homogeneous-property assumption shown to deviate locally by up to 67%
Zhu et al. [Zhu2026]	Base, weld and HAZ	E3	P	U	F	P	P	Physics-constrained neural constitutive model transferred between zones with limited additional data
Stutz et al. [Stutz2011]	Composite with FBG sensors	E3	P	P	F	P	P	Delamination analysis by numerical inversion of sensor data rather than a surface field
Chtourou et al. [Chtourou2017228]	Multi-steel spot weld	E3	U	U	F	U	U	Connector model identified; test configuration not specified in the extracted fields
Cognard et al. [Cognard2013]	Ductile adhesive	E3	F	U	F	U	U	Adhesive behaviour modelled at low temperature; full-field reporting not resolvable
Sun et al. [Sun20154358]	FSW Al-Cu (simulated)	E4	F	N	F	P	N	GTN parameters fitted to a simulated tensile test; no experimental field and no independent validation
Nayakanti and Subramanian [Nayakanti2013]	Idealised FGM (synthetic)	E4	F	N	F	P	N	Eigenfunction VFM recovers a spatially varying modulus from numerically generated fields; method verification, not experimental validation
Sakaguchi et al. [Sakaguchi2026Creep]	Notched IN718 creep specimen	E3	F	P	F	F	F	Full-field strain and force support creep-parameter identification and independent comparison with baseline uniaxial creep data; DIC resolution and noise limits are discussed but measurement uncertainty is not propagated quantitatively
Hamill et al. [Hamill2026]	Welded-joint property map (synthetic)	E4	F	F	F	F	N	Automated spatial elastoplastic mapping demonstrated on synthetic fields; no experimental validation

Study	Specimen	Tier	I1	I2	I3	I4	I5	Governing appraisal note
Schroeder et al. [Schroeder2026]	S690QL weld HAZ	E3	F	P	N	N	N	Local HAZ performance measured for microalloying variants; no inverse identification
Wang et al. [Wang2026HAZ]	High-strength-steel HAZ	E3	F	F	F	P	F	Multiscale local tests and modelling establish zone-resolved behaviour with independent comparisons
Yadav et al. [Yadav2025]	Friction-stirred Al–Cu–Li	E3	F	P	F	P	F	Local constitutive behaviour determined from experimental fields across an incidental process zone
Larichkin et al. [Larichkin2025]	Welded steel fracture specimens	E3	F	P	F	P	P	Temperature-dependent fracture data support nonlocal damage calibration; spatial WAAM transfer remains indirect
Jones et al. [Jones2024DIC]	304L multi-geometry data set	E3	F	F	N	N	N	Synchronous DIC and thermography data for seven geometries; reusable validation data, not an identification study
Yan et al. [Yan2023HAZ]	Cold-formed RHS weld HAZ	E3	F	P	F	P	F	Equivalent HAZ properties calibrated and checked in structural connection models
Que et al. [Que2023]	Undermatching X80 weld	E3	F	P	N	N	N	DIC resolves heterogeneous deformation and damage; no constitutive inversion
Stanisz et al. [Stanisz2022]	Joints made by different processes	E3	F	P	P	N	P	Process-dependent zones compared; identification and independent validation remain incomplete
Lattanzi et al. [Lattanzi2020]	Anisotropic sheet specimens	E3	F	F	F	F	F	Experimental non-linear VFM strategies compared for transformation-based anisotropic plasticity
Zhang et al. [Zhang2019PostNecking]	Thick high-strength steel	E3	F	F	F	F	F	Post-necking hardening recovered from full-field diffuse-necking measurements and validated numerically
Nimer et al. [Nimer2019]	Ti-5111 microtensile zones	E3	F	P	P	N	P	Local mechanical response and failure mechanisms compared at two microstructural scales; no spatial inversion
Jia et al. [Jia2017]	Laser-welded DP980	E3	F	P	F	P	F	Experimental and numerical local-property assessment links HAZ zones to failure
Milošević et al. [Milosevic2016]	Local zones of welded joints	E3	F	P	P	N	N	DIC maps local stiffness differences; no identified continuous constitutive law
Srinivasan et al. [Srinivasan2016]	Welded mild steel	E3	F	P	N	N	N	Zone-resolved thermomechanical yielding observed; parameters were not inversely identified
Bertin et al. [Bertin2016]	Cruciform specimen (synthetic fields)	E4	F	F	F	F	P	Geometry optimised for constitutive-parameter identifiability; synthetic verification precedes physical validation
Yue et al. [Yue2015]	Notched rolled-sheet specimens	E3	F	P	F	F	F	Hybrid experimental–numerical identification couples anisotropic plasticity and damage over several triaxialities
Khalfallah [Khalfallah2014]	Welded tubes	E3	F	N	F	P	F	Weld-bead and HAZ properties assessed experimentally and numerically for hydroforming; spatial resolution limited
Nakata and Tanigawa [Nakata2012]	F82H welded-joint fatigue specimen	E3	F	P	N	N	N	DIC evaluates local deformation accompanying fatigue damage; no constitutive identification
Rossi and Pierron [Rossi2012]	Large-deformation specimen (synthetic 3-D fields)	E4	F	N	F	P	N	VFM plastic-parameter recovery demonstrated from three-dimensional displacement fields without independent experiment

Two patterns carry directly into the synthesis. In Table S3, M1 is met throughout, whereas M4 is most often partly met or unclear; the mechanical claims therefore carry an M4 appraisal downgrade rather than a certainty based on study count alone. In Table S4, the tier-E1 rows are rated N on I3–I5, whereas the rows rated F on those domains are all tier E2–E4. The direct graded-manufacturing and inverse-identification capabilities are therefore disjoint in this corpus.
